# Evolutionary game and simulation study of public transport under government incentive and punishment mechanism

**DOI:** 10.1371/journal.pone.0311286

**Published:** 2024-10-01

**Authors:** Mingyue Chen, Chunyan Li

**Affiliations:** School of Management Engineering, Capital University of Economics and Business, Beijing, China; Stellenbosch University, SOUTH AFRICA

## Abstract

Public transport plays an indispensable role in the whole public transport system. This paper makes an in-depth study on how public transport can provide passengers with higher service quality while meeting the needs of passengers. In order to achieve this research goal, this paper organically incorporates the three key subjects of government supervision, public transport and passengers into the research framework. Evolutionary game theory is used to construct the corresponding research model. It has been found that the decision-making behaviours of government regulators, public transport and passengers are intricately intertwined to influence each other in the evolutionary process. It is particularly noteworthy that the incentive or punishment measures adopted by the government have a great impact on the quality of public transport services. In addition, timely supervision and inspection of government regulatory authorities by higher authorities proved to be crucial for buses to provide stable and high-quality services. This study reveals the mechanisms of interaction between different subjects in the public transport system, particularly the government-guided incentive measures and supervision mechanism to promote the overall service level. To further support the research conclusions, this paper carries on the simulation analysis, and puts forward the countermeasures and suggestions for the bus to provide high-quality service according to the simulation results. These recommendations will help guide the government, public transport and passengers to make better decisions in the synergistic development process, thereby improving the overall level of service.

## Introduction

With the flourishing development of the economy, urban transport systems are facing increasingly complex challenges and opportunities. While people’s living standards continue to improve, the demand for travelling is gradually showing a trend of personalisation and high quality. This trend is not only a test for the adaptability of urban transport systems, but also an urgent demand for the improvement of urban functions. Against this background, the rational and healthy development of the public transport system has become particularly important, and the key lies in how to better provide high-quality services for passengers. Researchers pay attention to this trend not only out of concern for the urban transport system, but also to promote the improvement of urban functions. How to make public transport more responsive to the actual needs of the public and improve the quality of service has become an urgent problem to be solved. There are a series of problems in the current public transport systems, of which the quality of service provided by public transport is one of the most prominent. In order to deeply solve this problem, we need to explore it from three key perspectives: government, bus company and passengers.

Firstly, governments play a crucial role in decision-making and planning in urban transport systems. Effective government intervention and rational planning are the basis to ensure that the public transport systems can adapt to people’s travel needs. Secondly, as the main body of transport, the level of management and operation strategies of public transport companies directly affect the quality of service. Finally, passengers are the ultimate beneficiaries of the public transport systems, and their needs and satisfaction directly measure the quality of public transport services. The role of the government in formulating more scientific and rational policies and planning, the responsibility of bus companies in improving operational efficiency and service levels, and the role of passengers in providing feedback and active participation are the core content of this study.

In order to make the model more universal, an evolutionary game model is established by thoroughly studying the three perspectives of the government, bus companies and passengers, with a special emphasis on the reaction of the evolutionary dynamics to the behaviours and decisions of all parties in the system. Evolutionary dynamics is used to reflect the variability of the urban transport environment and capture the dynamic changes in government policies, bus operations, passenger demand and other factors. Through a deep understanding of evolutionary dynamics, we can better reveal the complex relationships among the participants in the model, and then propose more practical and feasible cooperative combination strategy.

Through this research, not only helps to provide more specific suggestions and countermeasures for public transport, so that it can better provide high-quality services for passengers, but also provides useful theoretical support and practical suggestions for the sustainable development of the public transport system and the improvement of the efficiency of urban transport operation.

The rest of the paper is as follows: The Literature Review section provides the research of game modelling in the field of public transport, which is reflected in fare setting and revenue distribution, strategic decision analysis, route planning and competition, resource allocation and optimization, traffic congestion and right-of-way allocation, etc. The Evolutionary Game Model section, which formulates the hypotheses and constructs the problem description, basic assumptions, the construction of the three-party evolutionary game model; The Model Analysis section, based on the establishment of the tripartite evolutionary game model, this paper analyzes its strategic stability. In order to further confirm the research conclusions, The Simulation Analysis section includes a simulation analysis, and the main conclusions and further research directions are proposed in The Conclusions section.

The innovations of this paper are as follows: First, previous studies have mainly focused on the optimization of transit systems and route design, etc., while less consideration has been given to the impact of transit service quality on passengers’ travel choices and the overall size of the transit market. This paper looks at service quality and explores its key role in enhancing the attractiveness of public transportation.

Second, this paper introduces the government reward and punishment mechanism and constructs a three-party game model, highlighting the central role of the government in promoting high-quality public transportation services. In practical application, by adjusting the government reward and punishment mechanism, we can further assess the requirements of society and the government on bus service, so as to continuously optimize the service quality.

Thirdly, the effectiveness of the proposed gaming strategy is verified by introducing some actual data of China’s public transportation, simulating the evolution process of the public transportation system and analyzing it. Based on the relationship between the factors and the stabilization conditions, specific countermeasures and suggestions to promote the high-quality development of public transportation are proposed.

Fourth, this paper constructs an evolutionary game model framework that can more accurately reflect the interaction between transit operators and the government and the evolution of decision-making behavior. The framework is not only applicable to the bus system, but also can be extended to other transportation modes, creating a good public environment for the future development of public transportation.

## Literature review

Game theory plays an important role in public transport research, providing a theoretical framework for analyzing the interactions and decision-making between the various players in a transport system.

In terms of fare setting and revenue distribution, game theory can be used by scholars to analyze the fare setting and revenue distribution among the government, public transport operators and passengers, which is helpful to analyze the conflict of interest and cooperation of different subjects, so that the profitability of operators can be maintained, meet the needs of passengers, providing feasible suggestions for the sustainable development of public transport systems.

Based on the traffic congestion and parking problems, Wang et al [1] applied the principal-agent theory to the parking pricing problem to study the parking pricing problem by using the game theory method, and proposed a parking pricing model and established a parking pricing strategy. The parking pricing game involves three players, which are the government, drivers and parking companies. The private parking lot of Shanghai Metro Tower is selected as the object of analysis, and the previously mentioned parking pricing model is used for application analysis to demonstrate the reasonableness and applicability of the policy, and the results of the example analysis show that the pricing model can regulate the parking demand of drivers and alleviate the problems of traffic congestion and insufficient parking spaces. Gao et al [2] considered the problem of choosing the optimal service parameters by the carrier in public transportation traffic by building a two-stage game model, in the first stage, the carrier chooses the parameters of its service i.e. the number of vehicles, schedules etc. In the second stage, the participants announce the price of the service and the consumer chooses the appropriate service and an equilibrium is found in the pricing game, determining the central point of the model is the concept of rational consumer, assuming that after the announcement of the price of the service, the consumer chooses the service with the lowest cost in the sense of expectation, the cost includes the price of the service and the expected travel time, and ultimately, the optimal service parameters are determined as a solution to the non-cooperative game.

In the aspect of strategic decision analysis, game theory is used to analyze the strategic decisions among various players (e.g., government, operators, passengers, etc.) in public transport systems. Researchers can better understand the strategic choices of all parties by modeling and analyzing the interaction between decision makers. Zhu et al [3] explored the interaction between policy makers and travelers in the process of public transport policy making and proposed strategies to optimize the policy mix. Under the two cases of whether to set up bus-only lanes or not, considering the comfort of passengers in the car, the travel cost functions of buses and cars are established respectively, the behavior of travelers under different travel modes is revealed through the introduction of bottleneck model and public transport distribution flow model, and the Stackelberg game model between the government, public transport companies and travelers is established with the objective of minimizing the total cost of the system, the results show that under the assumption of travel distance of 20 km, the interaction between policy makers and travelers in the process of public transport policy making is not optimized. The results show that under the assumption that the travel distance is 20 km, the optimized strategy combination reduces the traffic safety cost by 8.59% and 9.82%, and the per capita travel cost by 10.28% and 15.85% under the two scenarios, respectively. Nie et al [4] applied carbon trading subsidy to the public transportation industry to study the interaction between the purchase decision of public transportation operators and the policy implementation decision of the government, and improved the evolutionary game theory to consider the incentive effect of strategies within the same group and discuss the stable strategies of each party. The correctness of the improved model is verified through simulation experiments, and the influence of key parameters on the evolution of decision-making behavior is investigated.

In terms of route planning and competition, game theory can be used by researchers to study the competition and cooperation between different public transport routes. By modeling the strategic choice of route planners, route allocation can be optimized and overall operation efficiency can be improved. Feng and others [5] established an optimization model of operating profit for a single bus line, which illustrated the importance of competition between bus lines in a bus network. Then, using the bus departure interval as the decision variable, the complete competition and imperfect competition models of two bus lines were established separately to maximize the operating profit of each line. Finally, a numerical example analysis was conducted using the two competition models to obtain the growth pattern of the operating profit of the two bus routes with the change of headway spacing, and to analyze the choice of the departure strategy of the two bus routes by using game theory and decision theory. It is found that there exists a law of increasing operating profit with the change of headway distance in competition for each route, and game theory and decision theory can be used to select their respective departure strategies. Liu and others [6] proposed a multidimensional Stackelberg game-based framework and mathematical model with the importance of the government, service providers, and passengers in planning new bus routes or adjusting existing routes. The model has a two-tier structure, with the upper tier reflecting the views of the government sector in the allocation of subsidies, and the lower tier reflecting the decisions of the service provider in the design of scheduling frequency and fleet size. The model is a Stackelberg game model in which the government agency plays the role of a leader and the service provider plays the role of a follower, with social costs and profits set as benefits, respectively. The bus route planning framework based on the Stackelberg game is able to reflect the decision-making sequence of the participants in bus route planning as well as the competitive or cooperative relationship among them. The impacts of these decisions on passengers’ transportation modes and route choices are analyzed through a Nested Logit model. Taking two parallel bus and subway lines in Harbin, China, as an example, Feng and others [7] used game theory to establish a utility model based on the frequencies of the two lines, and used Nash equilibrium to reveal and explain the root cause of the oversupply problem. To solve this problem, the authors propose a new operation model: the frequencies of the two modes are integrated to obtain a larger total profit, and then the total profit is redistributed to the two modes. The arithmetic example shows that this operation mode can effectively solve the oversupply problem while satisfying the demand of two operators, and the frequency of Nash equilibrium under the two modes is found to coincide with the actual operation after the study. Schiewe and others [8] took a new perspective on planning public transportation routes with the objective of minimizing the travel time of passengers, discussing the equilibrium of the game and the optimization methods for route planning and finding the relationship between the solutions, where the passengers are the participants, and the objective is to minimize the weighted sum of passenger travel time, transfer fines, and cost-sharing, under strong assumptions on the objective function, using the proposed best response algorithm to find the game equilibrium. Hu and others [9] studied the relationship between bus passengers’ path choice behavior and the game, analyzed the interaction between bus passengers’ path choice behavior and the game relationship, and the game benefits of bus passengers waiting for the platform were quantified by the number of passengers waiting for the platform, the number of passengers on the bus, the bus carrying capacity and other parameters, and constructed an evolutionary game model of the bus choice behavior under the condition of information induced, based on the characteristics of the passengers’ limited rationality, and analyzed the bus choice behavior under the condition of information induced. The bus choice behavior under the condition of information induction is analyzed, and through the evolutionary game model, the distribution of the proportion of passengers accepting the induction strategy when the evolution is stable is obtained, the passenger group behavior is dynamically adjusted, and suggestions for the implementation of the induction strategy are put forward.

In terms of traffic congestion and right-of-way allocation, game theory can be used to study the right-of-way allocation problem in urban traffic, and research scholars can analyse the game relationship between different traffic participants, so as to design a reasonable right-of-way allocation mechanism and reduce traffic congestion. Yao and others [10] studied the lane changing behavior of buses at stopping stations, used game theory to analyze the interaction between buses and drivers of social vehicles, established a two-person non-cooperative, non-zero-sum mixed-strategy game model, took into account the time saving and collision avoidance gains of the two sides of the game, and also took into account the characteristics of the buses’ large passenger load in the gains, and utilized the vehicle trajectory collected from the Dalian Green Wave Bridge Bus Harbor Station data, the model was calibrated using the great likelihood estimation method, and the validation results show that the formulated model can effectively predict the lane-changing decision with high accuracy. Among the mixed and dominant strategies, bus drivers are more inclined to choose the no lane-changing strategy, and social vehicle drivers are more inclined to choose the not giving way to buses strategy. The tendency of both participants to choose the strategy is consistent with the actual decision of drivers. Yao and others [11] explored the evolutionary dynamics of behavioral decisions of buses and social vehicles in different scenarios, and developed evolutionary game models for connected self-driving buses with social vehicles, human-driven buses with social vehicles, and connected self-driving buses with different penetration rates and social vehicles. This study applies evolutionary game theory to model the bus forced lane change exit problem. The equilibrium points of the dynamic game system are calculated based on differential equations constructed from the dynamics equations of replicators of different populations. The results of numerical experiments show that in most cases, connected autonomous buses choose to change lanes, while human-driven buses show a conservative trend in their strategies. Wu and others [12] proposed a collaborative prioritization method for transit signals that considers the game of transportation participants. The core of the collaborative prioritization method for bus signals is a bi-objective optimization problem with the objective of the desired delay of buses and the average delay of non-buses. And a finite state machine-based algorithm for estimating the average delay of non-bus vehicles is proposed by using game theory to achieve collaborative optimization instead of transforming the problem into a single-objective optimization problem through weighting. The randomness of bus arrival time is considered in the bus delay estimation to improve the robustness of the algorithm. And simulation experiments are conducted in Shanghai, China to verify the performance, and the results show that the bus delay can be effectively reduced under both low and high congestion. Koryagin and others [13] studied an urban passenger transport problem where municipalities and passengers are considered as participants in the passenger transport system, and municipalities have to optimize road widths and public transport frequency. Car travel time depends on the number of road lanes and the travel mode chosen by the passenger, who aims to minimize the total travel cost, including the value of time. Commuters try to find the optimal ratio between public transportation and automobiles, and the conflict between municipalities and commuters is described as a game model. The existence of a Nash equilibrium for the model is proved, and the effects of passenger flow timing and intensity on the equilibrium road width and public transport frequency are analyzed by means of an arithmetic example. Ding and others [14] proposed a game-based model for the study of road congestion charging problem, for example, the cost function of car or bus was introduced into the travel cost function of the traditional bottleneck model, based on which the Nash equilibrium between the government and different travelers was obtained based on the different travel cost functions of different modes of travel, and the results of the study can be used to describe the transportation system, respectively, the internalities and externalities. Finally, the conclusions are verified by two simulation examples.

Taking game theory as the basis for research in the field of public transportation as well as other aspects, Zhu and others [3] constructed a two-stage game model by introducing variety-seeking behavior and service level. In the first stage, the departure frequency of customized buses and public transportation is affected by service level, among others. In the second stage, the frequency of departure is only affected by the service level. Although the frequency of bus departures has an effect on the change of passenger flow rate in different stages, the effect of service level on passenger flow is significant. The results of the study show that the departure time or travel distance of buses deviates from the planned departure time or travel distance. Liu and others [15] proposed a bus waiting strategy based on dynamic control point importance ranking and selection for predicting headway based on cooperative game theory, which not only considered the influence of individual control points, but also the influence of collective control, the model consisted of a performance model and a cooperative game model, and the performance model predicted the headway performance of all the running buses with different combinations of control points, and the simulation experiments The results show that the algorithm can reduce the waiting time of passengers to a certain extent while reducing the variation of bus headway. Yao and others [16] studied the behavioral decision-making characteristics of drivers when buses are changing lanes down the lane, and analyzed the behavior between passenger car drivers and bus drivers with different driving styles in the target lane, and established a game model. The results show that considering driving styles helps to explore the interaction between passenger car and bus driver behaviors in depth.

Starting from a new perspective of dynamic evolutionary games, the authors consider the dynamic learning effect of commuters by modeling their travel mode choice behavior. Numerical experiments show that under a fixed parking fee policy, the evolutionary outcome is completely determined by travel time, and the only method of transit inducement is to increase the price of parking fees [17]. In assessing the impact of on-street parking on heterogeneous traffic operations on urban streets, the authors developed and calibrated a meta-cellular automaton model to simulate changing bike lanes on streets with on-street parking. Two street segments with different bike lane widths were considered, and through simulation, friction conflicts and blocking conflicts between bicycles and vehicles were identified, and the network impact of on-street parking on traffic assignment and operations was estimated [18]. On the basis of analyzing the residents’ low-carbon travel game and its evolutionary trend, the authors established a multidimensional game model of residents’ low-carbon travel. The game strategies in the two dimensions include whether to accept the low-carbon concept and whether to choose low-carbon traveling. Combined with the evolutionary game theory, the low-carbon travel choices of residents in different cities were simulated, and the evolutionary stability strategies of residents in different cities were obtained. The results of the study show that the proportion of accepting the low-carbon concept and choosing low-carbon travel is higher in cities with more developed public transportation systems [19].

In recent years, there has been a proliferation of new ways for commuters to use modes of transportation, and internet taxi is a more common way of getting around, but encounters territorial allocation problems, with most social cab drivers choosing areas where they are most likely to have the highest number of customers, and thus they negatively affect each other’s profits by offering a large number of services. The authors proposed a cooperative approach to territorial allocation, which can reduce conflicts between cab drivers through negotiation between service providers, and used game theory to formulate the territorial sharing problem, which can be solved using a bargaining-based solution model, and the results of the study were validated through the results of simulation work [20]. In spite of the default risk, online internet ridesharing services are becoming an important part of the public transportation system. The authors used the evolutionary game method to construct an evolutionary game model for internet ridesharing platforms, analyzed the equilibrium states of three scenarios, namely, no regulation, internal regulation by the platform enterprise, and external regulation by the regulator, and then simulated them. The results show that strong credit constraints or the establishment of a coordinated regulatory model with appropriate intensity are required to realize an evolutionary stability strategy for default risk control [21]. The emergence of online internet taxi services provides an innovative way of booking vehicles, but negatively affects the cab industry in China. The authors model cab service mode choice based on evolutionary game theory, with modes including dispatch mode and online taxi hailing mode. The results show that the adjustment of cab company behavior, driver behavior and passenger behavior affects the evolutionary path and convergence speed of cab company, driver and passenger behavior. However, it also reveals that the steady state in the game model remains the same regardless of the adjustments, and the findings provide a basis for studying the operation and management of cab systems [22]. The change of the net car industry has brought new challenges to the government governance, the authors introduce the coalition chain from the perspective of technology governance, construct the evolutionary game model of net car platform and the government under the blockchain technology, reveal the influence of the changes of the initial conditions and decision parameters on the evolutionary stability results by solving the replicated kinetic equations and Jacobi matrices, and carry out the numerical experiments by using the Python programming language. The results show that the additional cost of negative platform regulation and the government’s punishment have a positive effect on the evolution of the system to the ideal state. The cost of platform technology development and government innovation investment have a negative effect on the evolution of the system towards the ideal state [23]. The authors propose an evolutionary game-theoretic model for large-scale distributed renewable energy deployment in order to reveal how self-organized sustainable development of renewable energy in distribution networks can achieve low-carbon goals. In the evolutionary game, the return matrices of individual buses are built based on the load and renewable energy generation profiles, an evolutionary strategy is proposed based on the return matrices of individual buses and a dynamics model is derived for analyzing the penetration of renewable energy sources in distribution grids over multiple rounds of the evolutionary game [24].

In addition, school transportation services are of critical importance to society. The authors propose a new approach based on evolutionary games to reflect the “gaming” nature of this school transportation operational problem and to simulate the policy making process, which includes positive externalities. The paper considers two players, the school and the government. It is found that schools tend to be conservative and stop providing services if both parties are providing services, i.e. competing, and that schools and governments can jointly operate services in “equilibrium” and sustain this equilibrium if some key decision parameters such as profit thresholds can be changed [25]. The last-mile problem is a hot topic in transportation engineering, in which cabs, buses, and shared bikes are three different modes chosen by individuals that will have a significant impact on the personal utility of traveling passengers. Based on the characteristics of different travel modes, the authors establish an evolutionary game model for travel mode choice based on replicated kinetic equations, taking into account pedestrian perceptions and travel costs. By calculating the stable equilibrium state of the system under different cost parameters, the way of travel mode evolution in an infinite population is analyzed. The results show that when the system reaches a stable equilibrium state, the proportions of the three travel modes are very sensitive to changes in travel costs [26].

It can be seen that game theory provides researchers with a framework for analyzing various interrelationships in complex transportation systems, which helps to formulate more practical and feasible transportation policies and strategies. Through the application of game theory, the decision-making process in public transportation systems can be better understood and the efficiency and sustainability of the overall system can be improved.

## Evolutionary game model

### Problem description

In the urban transport system, public transport plays an important role in meeting the travelling needs of residents. However, in the process of providing services, public transport often faces a series of complex and serious problems. First of all, there are many stakeholders involved in the market, including the government, bus companies and passengers, and their different needs and interests make the operation of public transport systems face multiple challenges.

On the one hand, passengers expect bus systems to provide efficient, convenient and safe services to meet their diverse travelling needs. On the other hand, bus companies and governments are under pressure from operating costs, route planning, vehicle maintenance and other aspects, and need to ensure the economic viability of the system while safeguarding the quality of service. Currently, there are a number of problems with the quality of service of public transport systems. For example, unreasonable route planning makes it difficult for passengers in some areas to obtain timely and convenient services; Improper vehicle scheduling can result in congestion and inappropriate waiting times; and irrational fare strategies may affect the accessibility of services. These problems directly affect the overall operational effectiveness of public transport systems and user experience.

In order to solve these problems, this study aims to explore in depth the effective ways to improve the quality of public transport services while meeting the needs of passengers through the study of public transport game evolution. Specifically, this paper will establish an effective evolutionary model through the introduction of game theory, so that the stakeholders can form a dynamic equilibrium in the system, so as to promote the development of public transport system to the direction of higher quality services. The purpose of this paper is to deeply understand the problems in the public transport system, to further explore the relationship between the three parties through the game evolution model, and to propose solutions, so as to provide useful references for public transport managers, policy makers and relevant researchers, and to promote the development of the public transport system in the direction of more efficient.

The model construction of this paper refers to the following literature, in order to improve the efficiency of government regulation and promote the market application of electric buses, the authors established the electric bus production enterprises, passenger transport enterprises, and the government as the three-party subjects of the evolutionary game, and deduced equilibrium stabilization strategies through the analysis of game equilibrium and evolutionary stability, to quantitatively analyze the impact of changes in the key variables on the decision-making subject to enhance the behavioral choices [27]. In addition, the authors established a four-party evolutionary game model, considered the impacts of government, platforms, drivers and passengers on the regulation of the online car market, analyzed the strategic stability of the evolutionary game model, and verified the analysis results through simulation [28]. The safety supervision of online car pooling is of great significance for alleviating the pressure of urban public transportation and facilitating the people’s safe and convenient travel. Based on the tripartite evolutionary game theory, we describe the interaction mechanism of the government regulator, the safety supervision department of online car pooling platform, and the carpooling owner in the process of China’s online car pooling operation, and use the system dynamics method to explore the dynamic simulation process of the evolutionary game model [29]. By constructing a tripartite evolutionary game model of government, operators and users, the authors explore the evolutionary law of operators’ strategy choices by considering the combined effects of multiple factors [30]. The development of public transportation priority cannot be separated from the support of the government’s financial and tax policies, while the implementation of subsidy policy is the joint role of multi-interested parties. The authors analyze the implementation mechanism of public transport subsidy policy from the perspective of carbon emission, explore the evolutionary stabilization strategy of the three-party game under different circumstances, and clarify the key factors affecting the evolutionary path of the stabilization strategy through simulation analysis. The authors conclude that strengthening government regulation can help reduce the misrepresentation of public transport enterprises in the implementation of public transport subsidy policy, and the penalties and undue profits brought by misrepresentation are also key factors affecting the behavioral decisions of the government and enterprises [31].

**Fig 1:**
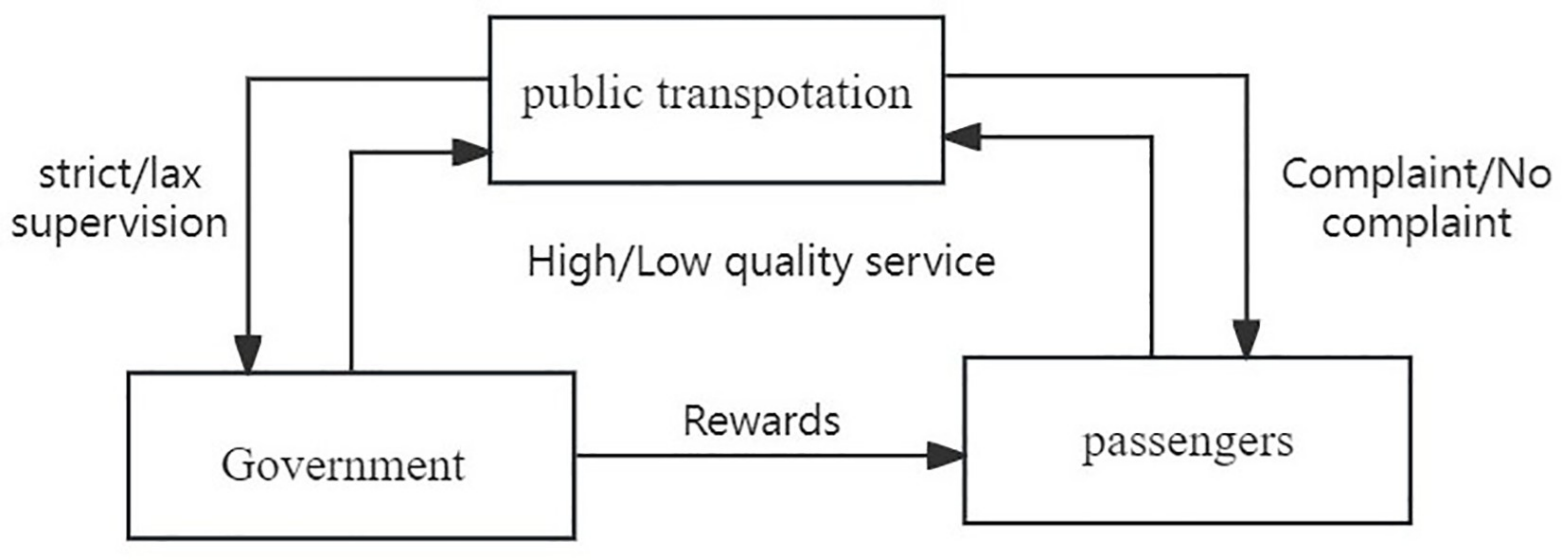
Tripartite evolutionary game logical relationship diagram.

To sum up, this paper builds a tripartite evolutionary game logical relationship diagram based on government regulators, public transport and passengers, as shown in Fig 1.

### Assumptions of game model

**Hypothesis 1:** Player 1 represents public transport, Player 2 represents travelling passengers, and Player 3 represents government regulator. It is assumed that the strategy choices of these three parties gradually evolves and stabilizes to the optimal strategy over time.

**Hypothesis 2:** Assuming that the strategy space for public transport is a = (a_1_,a_2_) that is (high service quality, and low service quality), choose a_1_ with the probability of x, choose a_2_ with a probability of (l-x),x∈[0,1]; and assume that the strategy space for commuting of travelling passengers is β = (β_1_,β_2_) that is (complain, not complain), choose β_1_ with the probability of y, choose β_2_ with a probability of (1-y), y∈[0,1]; and assume that the strategy space of the government regulatory is γ = (γ_1_γ_2_), that is (strict regulation, loose regulation), choose γ_1_ with a probability of z, choose γ_2_ with a probability of (1-z), z∈[0,1].

**Hypothesis 3:** Assume that the revenue of public transport is R_p_, the operating cost of public transport providing high-quality services to passengers is C_ph_, public transport against the rules and regulations for passengers to provide low-quality services to the social cost is C_pl_, In this case, where C_ph_>C_pl_. when the public transport for passengers to provide a better service, the order of public transport is stable, when the public transport for passengers to provide a low-quality service will affect the choice of mode of travel for passengers, to some extent, it will reduce the company’s earning is B_t_, where, B_t_<(C_ph_−C_pl_).

**Hypothesis 4:** When public transit provides low-quality services to passengers, passengers do not complain will give passengers a poorer experience, the cost is C_t_, and the service benefit obtained by passengers during the ride is V_t_.

**Hypothesis 5:** When the public transport provides low-quality service, passengers will complain to the government regulator, and the government regulator will fine the public transport when it is strictly regulates the service, the fine amount is F_p_, the government regulator will reward the passengers as M_t_, When the public transport provides high quality service, the government regulator will reward the public transport, The reward amount is M_p_, assuming that the cost of strict regulation by the government regulator is G_c_.

**Hypothesis 6:** When public transport provides high quality service to passengers, it brings some social benefits to the government regulator, denoted as R_g_. When public transport provides low quality service, it costs the government regulator to maintain public order, The cost is G_w_. If public transport provides low quality of service, when the government department carries out lax regulation, it will lead to social disorder and will be punished by the higher authorities, the penalty cost is T_g_,Where, T_g_>G_c_. The basic parameters of this paper are shown in Table 1.

**Table 1:** Parameter description.

Symbol	Meaning	Symbol	Meaning
R_p_	Bus revenue	F_p_	Government regulatory fines for buses
C_ph_	Costs for high-quality service	M_p_	Government regulatory rewards for buses
C_p1_	Costs for low-quality service	M_t_	Government regulatory rewards for passengers
B_t_	Reduced revenue	G_c_	Cost of strict government regulation
C_t_	Operating costs	R_g_	Benefits brought to government regulation
V_t_	Service received during the ride	G_w_	Costs by government regulatory
T_g_	Cost of penalties		

### Model construction

Based on the above assumptions, the mixed-strategy game matrix of public transport, travelling passengers and government regulators is shown in Table 2. The hypothetical mixed-strategy game matrix of public transit, traveling passengers, and government regulators is described in detail. When transit provides high-quality service with strict government regulation and passengers choose to complain, the overall benefit to transit is the benefit to transit, minus the cost it pays to provide high-quality service to passengers, plus the government regulator’s incentive to transit; the benefit to passengers is the service they receive in riding the bus; and the benefit to the government is the benefit from government regulation minus the cost to the government regulator of strict regulation, minus the government’s regulatory incentives for transit.

**Table 2:** Mixed strategy game matrix.

	passengers	Strict regulation Z	Lax Regulation 1−Z
**High service quality** **X**	Complaints y	R_p_−C_ph_+M_p_, V_t_,R_g_−G_c_−M_p_ ;	R_p_−C_ph_, V_t_, R_g_;
	No complaints 1−y	R_p_−C_pl_+M_p_, V_t_, R_g_−G_c_−M_p_;	R_p_−C_ph_, V_t_, R_g_;
**Low service quality** **1−x**	Complaints y	R_p_−F_p_−C_pl_−B_t_, V_t_+M_t_, −G_c_+F_p_−M_t_ ;	R_p_−C_pl_−B_t_, V_t_, 0;
	No complaints 1−y	R_p_−C_pl_−B_t_ V_t_−C_t_, −G_c_−G_w_;	R_p_−C_pl_−B_t_, V_t_−C_t_, −G_w_−T_g_;

When transit provides high-quality service, government regulates loosely, and riders choose to complain, the overall benefit to transit is the benefit to transit, net of the cost it pays to provide high-quality service to riders; the benefit to riders is the service they receive in riding the bus; and the benefit to the government is the benefit from government regulation.

When transit provides high-quality service with strict government regulation and passengers choose not to complain, the overall benefit to transit is the benefit to transit, minus the cost it pays to provide high-quality service to passengers, plus the incentive to transit from government regulators; the benefit to passengers is the service they receive in riding the bus; and the benefit to the government is the benefit from government regulation minus the cost to government regulators of strict regulation, minus the cost of government regulator’s incentives for transit.

When transit provides high-quality service, the government regulates loosely, and riders choose not to complain, the overall benefit to transit is the benefit to transit, net of the cost it pays to provide high-quality service to riders; the benefit to riders is the service they receive in riding the bus; and the benefit to the government is the benefit from government regulation.

When public transit provides low-quality service, the government strictly regulates it, and passengers choose to complain, the overall benefit to public transit is the benefit to transit, minus the fine imposed on transit by the government regulator, minus the cost of running the low-quality service, and minus the reduction in the benefit to transit; the benefit to the passenger is the service received in the ride, plus the incentive to the passenger imposed by the government regulator; and the benefit to the government sector is the fine imposed on transit by the government regulator, minus the cost of running the low-quality service, and minus the reduction in the benefit to transit. minus the cost of strict regulation by the government regulator, minus the incentives to riders by the government regulator.

When transit provides low-quality service, with lax government regulation and passengers choosing to complain, the overall benefit to transit is the benefit to transit, net of the cost of running the low-quality service and net of the reduced benefit to transit; the benefit to passengers is the service they receive in the ride; and the benefit to government is zero.

When public transit provides low-quality service, the government strictly regulates it, and passengers choose not to complain, the overall benefits of public transit are the benefits of public transit, net of the costs of running the low-quality service, and net of the reduced benefits of public transit; the benefits of passengers are the services they receive in the rides, net of the social costs of the low-quality service; and the benefits of the government sector are the costs of strict regulation by the government regulator, net of the cost of the costs incurred by the government regulator.

When public transit provides low-quality services, the government regulates loosely, and passengers choose not to complain, the overall benefit to public transit is the service received in the ride, minus the social cost of the low-quality service; the benefit to passengers is the service received in the ride, minus the social cost of the low-quality service; and the benefit to the government sector is the cost of the strict regulation by the government regulator, minus the cost of the penalties imposed on the government’s lax regulation.

## Model analysis

### Strategic stability analysis of public transport

The expected revenue and average expected revenue (E_11_,E_12_, $\bar{E_{1}}$) of public transport providing high or low quality service are:

$$\begin{array}{l} E_{11}=y\times z\times (R_{p}-C_{ph}+M_{P})+y(1-z)\times (R_{p}-C_{ph})+z\times (1-y)\\ \times (R_{p}-C_{ph}+M_{p})+(1-y)(1-z)\times (R_{p}-C_{ph})\\ E_{12}=y\times z\times (R_{p}-C_{pl}-F_{p}-B_{t})+y\times (1-z)\times (R_{p}-C_{pl}-B_{t})\\ +z\times (1-y)\times (R_{p}-C_{pl}-B_{t})+(1-y)\times (1-z)\times (R_{p}-C_{pl}-B_{t})\\ \bar{E_{1}}=xE_{11}+(1-x)E_{12}=x\times (B_{t}-C_{ph}+C_{pl})+R_{p}-C_{pl}-B_{t}\\ -F_{p}\times y\times z+M_{p}\times x\times z+F_{p}\times x\times y\times z \end{array}.$$ (1)

The replication dynamic equation for public transport strategy selection is as follows:

$$F(x)=dx/dt=x(E_{11}-\bar{E_{1}})=-x(x-1)\times (Bt-C_{ph}+C_{pl}+M_{p}\times z+F_{p}\times y\times z)$$ (2)

The first-order derivatives of x and the set G(y) are respectively:

$$x\frac{d(F(x))}{dx}=(-2x+1)\times (Bt-C_{ph}+C_{pl}+M_{p}\times z+F_{p}\times y\times z).$$ (3)

$$G(y)=Bt-C_{ph}+C_{pl}+M_{p}\times z+F_{p}\times y\times z$$ (4)

According to the stability theorem of differential equations, the probability that the public transport chooses high-quality service in a stable state must satisfy:

F(x) = 0 and d(F(x))/dx<0x = 0. Due to ∂G(y)/∂(y)>0, So G(y) with respect to y is an increasing function.

**Fig 2:**
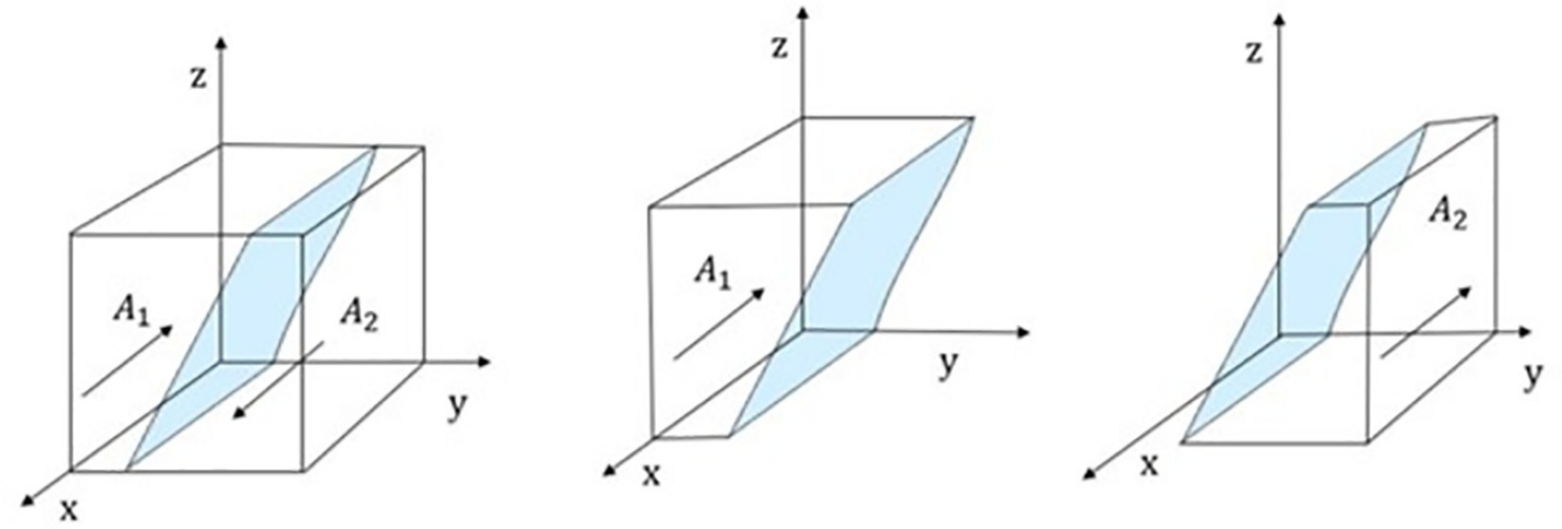
Phase diagram of strategy evolution for buses.

Therefore, when y = (B_t_−C_ph_+C_pl_+M_p_×z)/(−z×F_p_) = y*,G(y) = 0,In this case,d(F(x))/dx = 0, The public transport does not determine the stability strategy; when y>y*,G(y)>0,In this situation, d(F(x))/dx|_x = 1_<0,x = 1 is the evolutionarily stable strategy of public transport. (Evolutionarily Stable Strategy, ESS); Conversely, is an Evolutionarily Stable Strategy (ESS). The phase diagram of public transport strategy evolution is shown in Fig 2.

As shown in Fig 2, the probability of public transport providing stable low-quality service to passengers is volume $V_{A_{1}}$ of A_1_, and the probability of public transport providing stable high-quality service to passengers is volume $V_{A_{2}}$ of A_2_, which can be calculated as follows:

$$V_{A_{1}}=\int_{0}^{1}\int_{0}^{1}\frac{B_{t}-C_{ph}+C_{pl}-R_{g}+M_{p}\times z}{-z\times F_{p}}dzdx=\frac{‐M_{p}}{F_{p}}$$

$$V_{A_{2}}=1-V_{A_{1}}=\frac{M_{P}+F_{p}}{F_{p}}$$

**Theorem 1:** The probability of the bus providing high-quality service to passengers is positively correlated with the bus operating revenue and government rewards and penalties, and negatively correlated with the cost of the bus providing low-quality service to passengers.

Prove: According to the probability expression of public transport to provide high-quality service, the first order partial derivatives of each element are obtained:

$\partial V_{A_{2}}/\partial (F_{P}+M_{p})>0$, Therefore, when public transport provides high-quality service, increase M_p_, when public transport provides low-quality service, also increase F_p_, can make the probability of public transport to provide high-quality service increase.

The result suggest: When the public transport provides high-quality services, the government regulator can reward the public transport by taking incentive measures. When the public transport provides low-quality services, the government regulator can not only increase the punishment, but also increase the cost of providing low-quality services through the supervision of the media and the public, so as to promote the public transport to provide high-quality services for passengers.

**Theorem 2:** In the evolution process, the probability of public transport providing high-quality services will rise with the increase of travelling passenger complaints and strict regulation by government regulators.

Prove: From the analysis of the strategic stability of public transport:

When z>C_ph_−C_pl_−B_t_/M_p_+y×F_p_,_y<y*_,G(y)>0,d(F(x))/dx|_x = 0_<0, Then x = 1 is the evolutionary equilibrium strategy; conversely, x = 0 is the evolutionarily stable strategy.

Therefore, as y and z gradually increase, the stability strategy of public transport increases from x = 0(providing low quality service) to x = 1 (providing high quality service).

The result suggest: Increasing the probability of passengers complaining about public transport will be help public transport choose to provide high-quality services as a stable strategy. The government regulator can not only increase the probability of strict regulation by the government to ensure that public transport provides a high quality of service, but also reward passengers who complain through passenger complaints and increase their sense of social responsibility. Give full play to the various subjects in the society to supervise the service quality provided by public transport, so as to build a harmonious and stable public transport order and promote a virtuous cycle.

### Analysis of the strategic stability of traveling passengers

The expected revenue of travelling passenger complaints or non-complaints and the average expected revenue (E_21_,E_22_, $\bar{E_{2}}$) are respectively:

$$\begin{array}{l} E_{21}=x\times z\times V_{t}+x\times (1-z)\times V_{t}+(1-x)\times z\times (V_{t}+M_{t})+(1-x)\times (1-z)\times V_{t}\\ E_{22}=x\times z\times V_{t}+x\times (1-z)\times V_{t}+z\times (1-x)\times (V_{t}-C_{t})+(1-x)\times (1-z)\times (V_{t}-C_{t})\\ \bar{E_{2}}=xE_{21}+(1-x)E_{22}=V_{t}-C_{t}+x\times C_{t}+y\times C_{t}-x\times y\times C_{t}+y\times z\times M_{t}-x\times y\times z\times M_{t} \end{array}$$ (5)

The replicated dynamic equation for passenger strategy selection is as follows:

$$F(y)=dy/dt=y(E_{21}-\bar{E_{2}})=y\times (C_{t}+M_{t}\times z)\times (x-1)\times (y-1)$$ (6)

$$\frac{d(F(y))}{dy}=(x-1)\times (2y-1)\times (C_{t}+M_{t}+z)$$ (7)

$$J(z)=(x-1)\times (C_{t}+M_{t}+z)$$ (8)

According to the stability theorem of differential equation, the probability of passengers choosing to complain in a stable state must be satisfied:

F(y) = 0 and d(F(y))/dy<0.Due to ∂J(z)/J(z)>0,the function J(z) with respect to z is a increasing function.

**Fig 3:**
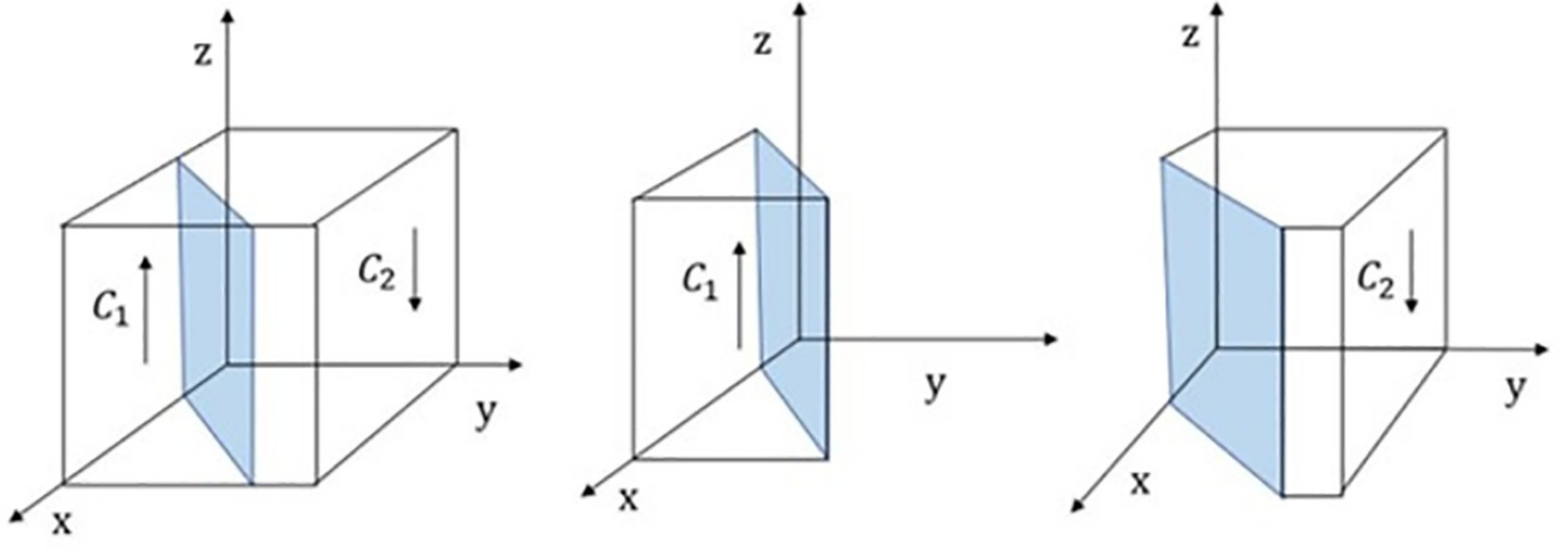
Phase diagram of passenger strategy evolution.< /Figure_Caption>

Therefore, when z = −C_t_−M_t_ = z*,J(z) = 0, In this case, d(F(y))/dy = 0,F(y) = 0, the passenger cannot determine the stable strategy; when z<z*,J(z)<0,In this case, d(F(y))/dy|_y = 0_<0,y = 0 is the Evolutionarily Stable Strategy (ESS) of passengers; Conversely, y = 1 is ESS. The phase diagram of passenger policy evolution is shown in Fig 3.

Fig 3 shows that the probability that a passenger does not complain is the volume $V_{B_{1}}$ of B_1_and the probability that a passenger chooses to complain is the volume $V_{B_{2}}$ of B_2_, which is calculated as: $V_{B_{2}}=\int_{0}^{1}\int_{0}^{1}-C_{t}-M_{t}dxdy=-C_{t}-M_{t}$.

**Theorem 3:** The probability of travelling passenger complaints is negatively correlated with the benefits of low-quality service provided by public transport, and positively correlated with the punishment of public transport and the amount of reward given to passengers by government regulators. According to the expression of the probability of passenger complaint $V_{B_{2}}$, the increase of M_t_ can increase the probability of bus providing high-quality service.

The result suggest: When the public transport for passengers to provide low-quality services, travelling passengers will increase the probability of complaints. At this time, the government should give certain rewards to passengers and strictly supervise the public transport. In addition, the public transport drivers, conductors and ticket inspectors should also be trained to improve their professional qualities, and the media should expand the intensity of disclosure and other ways to promote their service quality.

**Theorem 4:** The non-complaint behavior of passengers in the evolution process will increase with the strict regulation rate of the government regulators or the increase of the probability that the public transport provides high-quality service.

The result suggest: The probability of strict regulation by government regulators and the probability of high-quality service provided by public transport can both induce travelling passengers to reduce their complaints against public transport as a stabilising strategy.

### Analysis of strategic stability of government regulatory

The expected income and average expected income (E_31_,E_32_, $\bar{E_{3}}$) of strict or loose regulation by government regulators are respectively:

$$\begin{array}{l} E_{31}=y\times x\times (-G_{c}-M_{p}+R_{g})+x\times (1-y)\times (-G_{c}-M_{p}+R_{g})+y\times (1-x)\\ \times (-G_{c}+F_{p}-M_{t})+(1-x)\times (1-y)\times (-G_{x}-G_{w})\\ E_{32}=x\times y\times R_{g}+x\times (1-y)\times R_{g}+y\times (1-x)\times 0+(1-x)\times (1-y)\times (-Tg-Gw)\\ \bar{E_{3}}=z\times E_{31}+(1-z)\times E_{32}=(z-1)\times [x\times (y-1)\times R_{g}+(x-1)\times (y-1)\\ \times (G_{w}+T_{g})-R\times x\times y]+z\times [y\times (y-1)\times (G_{c}-F_{p}+M_{t})+x\times (y-1)\times \\ \left(G_{c}-R_{g}+M_{p})-(x-1)\times (y-1)\times (G_{c}+G_{w})\right] \end{array}$$ (9)

$$\begin{array}{l} F(z)=dz/dt=z(E_{31}-\bar{E_{3}})=z\times (z-1)\times [G_{c}-T_{g}-y\times (F_{p}+M_{t}\\ +T_{g})+(M_{p}+T_{g})\times x+(F_{p}-M_{t}-T_{g})\times x\times y] \end{array}$$ (10)

$$\frac{d(F(z))}{dz}=(2z-1)\times [G_{c}-T_{g}-y\times (F_{p}+M_{t}+T_{g})+(M_{p}+T_{g})\times x+(F_{p}-M_{t}-T_{g})\times x\times y]$$ (11)

$$H(y)=(G_{c}-T_{g}-y)\times (F_{p}+M_{t}+T_{g})+(M_{p}+T_{g})\times x+(F_{p}-M_{t}-T_{g})\times x\times y$$ (12)

According to the stability theorem of differential equations, government regulator in a stable state must meet:

F(z) = 0 and d(F(z))/dz<0.Due to ∂H(y)/∂(y)<0,therefore, H(y) with respect to y is a decreasing function.

**Fig 4:**
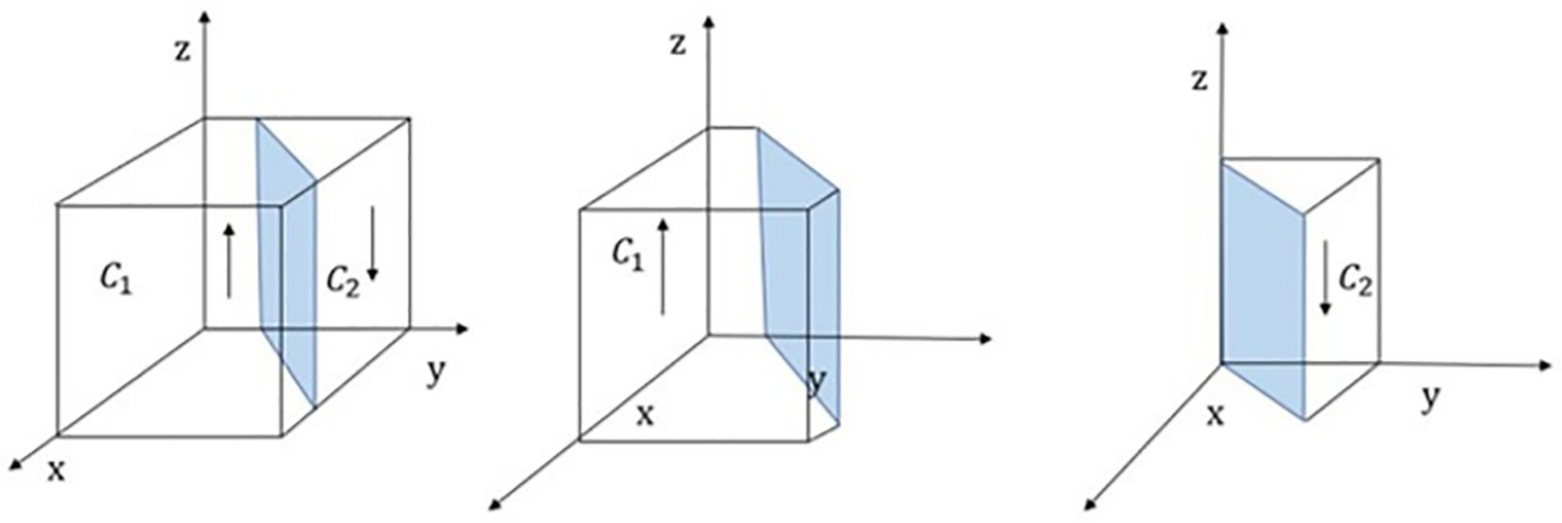
Phase diagram of government regulatory department strategy evolution.

When y = (G_c_−T_g_)×(F_p_+M_t_+T_g_)+×(T_g_+M_p_)/(x+1)×(F_p_+M_t_+T_g_) = y*,H(y) = 0,At this point, d(F(z))/dx = 0,The government regulatory department cannot determine a stable strategy; When y<y*,H(y)>0,At this point, d(F(z))/dz|_z = 1_<0,z = 0 is the Evolutionarily Stable Strategy (ESS) of the government supervision department; Conversely,z = 1 is ESS. The strategy evolution phase diagram of government regulator is shown in Fig 4.

Fig 4 shows that the probability of strict regulation by the government regulator is C_1_, with volume $V_{C_{1}}$, and the probability of loose regulation by the government regulator is C_2_, with volume $V_{C_{2}}$, which is calculated as follows:

$$V_{C_{1}}=\int_{0}^{1}\int_{0}^{1}\frac{\left(G_{c}-T_{g})\times (F_{p}+M_{t}+T_{g})+x\times (T_{g}+M_{p}\right)}{\left(x+1)\times (F_{p}+M_{t}+T_{g}\right)}dxdz$$

$$=\ln\frac{1}{2}\times (G_{c}-T_{g})+(1-\ln2)\times \frac{T_{g}+M_{p}}{F_{p}+M_{t}+T_{g}}$$

$$V_{C_{2}}=1-\ln\frac{1}{2}\times (G_{c}-T_{g})+(1-\ln2)\times \frac{T_{g}+M_{p}}{F_{p}+M_{t}+T_{g}}$$

**Theorem 5:** The probability of being strictly regulated by a government regulator is positively related to fines imposed by the government for providing low-quality service on public transport, rewards imposed by the government department for providing high-quality service on public transport, and rewards imposed on travelling passengers for complaining about the service, and is negatively related to penalties imposed by a superior.

Proof: According to the expression for the probability of strict regulation by the government regulator, the first partial derivative of each element is obtained:

$\partial V_{C_{1}}/\partial (T_{g}+M_{p})>0$, $\partial V_{C_{1}}/\partial (G_{c}-T_{g})>0$, $\partial V_{C_{1}}/\partial (F_{p}+M_{t}+T_{g})>0$, Therefore, An increase in M_t_、T_g_ or a decrease in G_c_−T_g_ cost can both increase the probability of strict regulation by the government regulator.

The result suggest: The higher the amount of punishment for public transport set by the government regulator, the more it will promote the government regulator to strictly supervise it, and conversely, if the amount of punishment for public transport set by the government regulator is lower, it will reduce the probability that the government regulator will strictly supervise it. In addition, a heavier penalty from the higher level of the government regulator will prompt the government regulator to strictly supervise the public transport, the higher the probability of strict government supervision, the higher the probability of travelling passengers not complaining about the public transport, which will help to stabilize the healthy and sustainable development of the public transport.

**Theorem 6:** Strict regulation by government regulators during the evolutionary process decreases as the probability of public transport providing high quality service or the number of non-complaints by passengers increases.

Proof: According to the strategic stability analysis of government regulatory departments, when x<(G_c_−T_g_−y)×(F_p_−M_t_−T_g_)/(−T_g_−M_p_−y)×(F_p_−M_t_−T_g_), z = 0 is the evolutionary equilibrium strategy; therefore, as y and x gradually increase, the stabilisation strategy of the government regulator increases from z = 0 (loose regulation) to z = 1 (strict regulation).

The result suggest: The rate of strict regulation by government regulators is influenced by the strategic choices of public transport and travelling passengers, and that when the probability of public transport providing high quality services or the probability of travelling passengers reducing their complaints is higher, government regulators will reduce their rate of strict regulation, but they will also lead to a lack of regulation.

### Stability analysis

The following system equilibrium can be obtained from F(x) = 0,F(y) = 0,F(z) = 0as follows:

$$\begin{array}{l} E_{1}(0,0,0),E_{2}(1,0,0),E_{3}(0,1,0),E_{4}(0,0,1),E_{5}(1,1,0),E_{6}(1,0,1),E_{7}(0,1,1),E_{8}(1,1,1)\\ E_{9}(0,‐(G_{c}‐T_{g})/(M_{t}‐F_{p}+T_{g}),‐C_{t}/M_{t})\\ E_{10}[‐(G_{c}‐F_{p}+M_{t})/(F_{p}+M_{p}‐M_{t}),1,‐(B_{t}‐C_{ph}+C_{pl})/(F_{p}+M_{p})]\\ E_{11}[‐(B_{t}\times {M_{t}}^{2}-C_{ph}\times {M_{t}}^{2}+C_{pl}\times {M_{t}}^{2}+C_{t}\times F_{p}\times G_{c}-B_{t}\times F_{p}\times M_{t}+C_{t}\times F_{p}\times M_{p}+C_{ph}\\ \times F_{p}\times M_{t}-C_{pl}\times F_{p}\times M_{t}-C_{t}\times F_{p}\times T_{g}-C_{t}\times M_{p}\times M_{t}+B_{t}\times M_{t}\times T_{g}-C_{t}\times M_{p}\times T_{g}-C_{ph}\\ \times M_{t}\times T_{g}+C_{pl}\times M_{t}\times T_{g})/(C_{ph}\times {M_{t}}^{2}-B_{t}\times {M_{t}}^{2}-C_{pl}\times {M_{t}}^{2}+B_{t}\times F_{p}\times M_{t}-C_{ph}\times F_{p}\times M_{t}\\ +C_{pl}\times F_{p}\times M_{t}+C_{t}\times F_{p}\times T_{g}+C_{t}\times M_{p}\times M_{t}-B_{t}\times M_{t}\times T_{g}+C_{t}\times M_{p}\times T_{g}+C_{ph}\times M_{t}\times T_{g}\\ -C_{pl}\times M_{t}\times T_{g}),(B_{t}\times M_{t}-C_{t}\times M_{p}-C_{ph}\times M_{t}+C_{pl}\times M_{t})/(C_{t}\times F_{p}),-C_{t}/M_{t}]\\ E_{12}[‐(G_{c}‐T_{g})/(M_{p}+T_{g}),0,‐(B_{t}‐C_{ph}+C_{pl})/M_{p}]\\ E_{13}[1,‐(B_{t}‐C_{\mathrm{ph}}+C_{\mathrm{pl}}+M_{p})/F_{p},1] \end{array}$$

According to the analysis, 13 pure strategy equilibrium points are obtained. Since x,y,z∈[0,1], the equilibrium point is meaningful under certain conditions, and the Jacobian matrix of the tripartite evolutionary game system is:

$$J=(\begin{array}{ccc} J_{11} & J_{12} & J_{13}\\ J_{21} & J_{22} & J_{23}\\ J_{31} & J_{32} & J_{33} \end{array})=(\begin{array}{ccc} \partial F(x)/\partial x & \partial F(x)/\partial y & \partial F(x)/\partial z\\ \partial F(y)/\partial x & \partial F(y)/\partial y & \partial F(y)/\partial z\\ \partial F(z)/\partial x & \partial F(z)/\partial y & \partial F(z)/\partial z \end{array})=$$

$$(\begin{array}{ccc} -x\times (B_{t}-C_{ph}\ldots ) & -F_{p}\times x\times z\times \ldots , & -x\times (M_{p}+F_{p}\times y)\ldots \\ y\times (C_{t}+M_{t}\times z)\ldots & y\times (C_{t}+M_{t}\times z)\ldots , & M_{t}\times y\times (x-1)\ldots \\ z\times (z-1)\times (M_{p}+\ldots ) & -z\times (z-1)\times (F_{p}-\ldots ), & \left(z-1)\times (G_{c}-T_{g}\ldots \right) \end{array})=$$

$$\left(\begin{array}{ccc} \begin{array}{l} -x\times \left(\begin{array}{l} B_{t}-C_{ph}+C_{pl}\\ +M_{p}\times z+F_{p}\times y\times z \end{array}\right)\\ -(x-1)\times \left(\begin{array}{l} B_{t}-C_{ph}+C_{pl}\\ +M_{p}\times z+F_{p}\times y\times z \end{array}\right) \end{array} & -F_{p}\times x\times z\times (x-1), & -x\times (M_{p}+F_{p}\times y)\times (x-1)\\ y\times (C_{t}+M_{t}\times z)\times (y-1) & \begin{array}{l} y\times (C_{t}+M_{t}\times z)\times (x-1)+\\ (C_{t}+M_{t}\times z)\times (x-1)\times (y-1), \end{array} & M_{t}\times y\times (x-1)\times (y-1)\\ z\times (z-1)\times (\begin{array}{l} M_{p}+T_{g}+F_{p}\times y\\ -M_{t}\times y-T_{g}\times y \end{array}) & \begin{array}{l} -z\times (z-1)\\ \times \left(\begin{array}{l} F_{p}-M_{t}-T_{g}-F_{p}\times x\\ +M_{t}\times x+T_{g}\times x \end{array}\right), \end{array} & \begin{array}{l} (z-1)\times \left(\begin{array}{l} G_{c}-T_{g}-F_{p}\times y+M_{p}\times x+M_{t}\times y+T_{g}\times x\\ +T_{g}\times y+F_{p}\times x\times y-M_{t}\times x\times y-T_{g}\times x\times y \end{array}\right)+\\ z\times \left(\begin{array}{l} G_{c}-T_{g}-F_{p}\times y+M_{p}\times x+M_{t}\times y+T_{g}\times x+T_{g}\times y\\ +F_{p}\times x\times y-M_{t}\times x\times y-T_{g}\times x\times y \end{array}\right) \end{array} \end{array}\right)$$

Using Lyapunov’s first method: all eigenvalues of the Jacobian matrix have negative real parts, then the equilibrium point is asymptotically stable; If at least one of the eigenvalues of the Jacobian matrix has a positive real part, the equilibrium point is an unstable point. In addition to the eigenvalue of the real part is zero, the other eigenvalues of the Jacobian matrix have negative real parts, then the equilibrium point is in a critical state, and the stability cannot be determined by the eigenvalue sign. The stability of each equilibrium point is analyzed, as shown in Table 3.

**Table 3:** Stability analysis of equilibrium points.

Equilibrium points	Eigenvalues of the Jacobian matrix		Stability conclusions
	*λ*_1_,*λ*_2_,*λ*_3_	Sign of the real parts	
**E**_**1**_ **(0,0,0)**	C_t_,T_g_−G_c_,B_t_−C_ph_+C_pl_	(+, +, -)	Unstable
**E**_**2**_ **(1,0,0)**	0,−G_c_−M_p_,C_ph_−B_t_−C_pl_	(0, -, +)	uncertain points
**E**_**3**_ **(0,1,0)**	V_t_,F_p_−G_c_−M_t_,C_p_−B_t_−C_ph_+C_pl_+R_p_	(+, +, +)	Unstable
**E**_**4**_ **(0,0,1)**	G_c_−T_g_,−C_t_−M_t_,B_t_−C_ph_+C_pl_+M_p_	(-, -, -) ^b^	ESS
**E**_**5**_ **(1,1,0)**	0,−G_c_−M_p_,C_ph_−B_t_−C_pl_	(0, -, +)	uncertain points
**E**_**6**_ **(1,0,1)**	0,−G_c_−M_p_,C_ph_−B_t_−C_pl_−M_p_	(0, -, -)	uncertain points
**E**_**7**_ **(0,1,1)**	−C_t_−M_t_,G_c_−F_p_+M_t_,B_t_−C_ph_+C_pl_+F_p_+M_p_	(-, -, -) ^b^	ESS
**E**_**8**_ **(1,1,1)**	0,−G_c_−M_p_,C_ph_−B_t_−C_pl_−F_p_−M_p_	(0, -, -)	uncertain points
**E**_**9**_	(−C_t_×M_t_×(G_c_−T_g_)..,B_t_×M_t_^2^..,C_t_×M_t_×(G_c_−T_g_)..	(/, /, /) ^a^	uncertain points
**E**_**10**_	−(F_p_+M_p_)…,(F_p_+M_p_)×(G_c_+M_p_)…,−Ct×M_p_^2^…	(/, /, /) ^a^	uncertain points
**E**_**11**_	(i, j, k) ^c^	(/, /, /) ^a^	uncertain points
**E**_**12**_	C_t_×M_p_^2^…,−M_p_×(M_p_+T_g_)..,M_p_×(M_p_+Tg)..	(/, /, /) ^a^	uncertain points
**E**_**13**_	0,0,G_c_+M_p_	(0, 0, +)	uncertain points

^a^ (/,/,/) indicates that the symbol cannot be determined.

^b^(-,-,-) are the coordinates of the corresponding equilibrium point, if the corresponding conditions of the equilibrium point are not met, the equilibrium point is unstable or meaningless. ESS stands for Evolutionary Stabilization Strategy.

^c^(i, j, k) represent three different formulas respectively, Due to the length of the formula, it is omitted here.

**Theorem 7:** Table 3 lists 13 equilibrium points. As can be seen from Table 3, When $G_{c}‐T_{g}<0,-C_{t}‐M_{t},B_{t}‐C_{ph}+C_{pl}+M_{p}<0,G_{c}‐F_{p}+M_{t}<0,B_{t}‐C_{ph}+C_{pl}+F_{p}+M_{p}<0$, the replicator dynamic system has stable points where *E*_4_(0,0,1) *E*_7_(0,1,1).

The result suggest: When the number of passenger complaints is low or the punishment of the government regulator is small, the probability of public transport providing high-quality service is relatively low, and according to the difference of the initial point chosen by the three-party measurement, the strategy combinations evolve and stabilise at (public transport provides low-quality service, passengers don’t complain, and it is strictly regulated) and (public transport provides low-quality service, passengers complain, and the government strictly regulates it). At this point, even if the government regulates strictly but the punishment is small, it is not able to restrain x1, x2, x3, y1, y2, y3, z1, z2, z3 the public transport effectively, and the public transport cannot provide high-quality service for the passengers. Therefore, in order to avoid the emergence of the stable strategy combinations, the government regulator should set a larger amount of fines or incentives, and give full play to the effectiveness of the reward and punishment mechanism of the government regulator.

## Simulation analysis

In order to verify the validity of the evolutionary stability analysis, the model was assigned numerical values for simulation analysis.

Array 1: R_p_ = 200, C_ph_-C_Pl_ = 85, C_p_ = 10, B_t_ = 40, F_p_ = 40, M_p_ = 30, C_t_ = 10, M_t_ = 25, G_c_ = 20, T_g_ = 40; where the transit revenue (R_p_ = 200) is the total revenue earned by the transit system under normal operating conditions, usually determined by fare revenue and ridership. This value represents the average revenue that a transit system can earn under normal operation. 200 This value is a reasonable level calculated based on a combination of factors such as passenger volume and transit fares. This parameter is usually referenced to the average daily actual operating data of local transit systems in China, such as fares, average daily ridership, and operating costs. Cost of High Quality Service (C_ph_)—Cost of Low Quality Service (C_pl_) = 85 indicates the additional cost of providing high quality service over low quality service, which may include vehicle maintenance, facility upgrades, staff training, etc. The difference of 85 is therefore a reasonable level of the cost of providing high quality service over low quality service. Therefore, this difference of 85 is an estimate based on historical operating data, budget reports, and equipment upgrade costs, and this value is usually derived from comparing actual cost differences under different quality levels of service.

Stochastic cost (C_p_ = 10) refers to unforeseen expenses or fluctuating costs during operation, such as expenditures for ad hoc maintenance, fuel price fluctuations, or other unexpected events. This parameter simulates the impact of uncertainties in actual operations. This value is estimated based on past ranges of cost fluctuations. Transit Reduced Revenue (B_t_ = 40): Lost revenue triggered by reduced ridership due to poor service quality or other factors, which is usually directly related to passenger satisfaction and willingness to choose transit. This value is estimated based on actual transit operations. For example, a reasonable value is estimated by counting changes in ridership and revenue loss when service quality changes. Government fine on public transportation (F_p_ = 40): the amount of penalty imposed by the government if the public transportation system fails to meet the service quality standards set by the government, this parameter aims to motivate the public transportation system to improve its service quality through economic means. This value is set according to the actual situation, and in this paper, it is set to 40 based on a combination of revenue and other factors. government incentives for public transportation (M_p_ = 30): the amount of incentives or rewards provided by the government when the public transportation system meets or exceeds the expected quality of service. Government incentives are usually set to motivate public transportation companies to improve service quality, and the value of 30 is an assessment of the actual incentive effect.

Social cost of public transportation to improve low-quality service (C_t_ = 10): the negative impacts and costs to society as a whole when the public transportation system provides low-quality service, such as increased passenger travel time, reduced travel efficiency, and increased pollution. This value is based on a comprehensive assessment and analysis of the parameters set in this paper. Government incentives to passengers (M_t_ = 25): government subsidies or incentives to incentivize more passengers to choose public transport, such as fare concessions, travel subsidies, etc., which are designed to enhance the overall attractiveness of the public transport market.25 This value is based on the analysis of a comprehensive assessment based on factors such as the amount of subsidies in the policy, as well as the parameters set in this paper. The cost of strict regulation by government departments (G_c_ = 20) refers to the fact that strict regulation requires human, technical, and institutional support, and the parameter value should come from the assessment of the relationship between regulatory intensity and cost inputs, but because this paper is more difficult to access within the government, the value of 20 is set in this paper based on the comprehensive assessment and analysis of the other parameters set in this paper. Penalties for lax regulation (T_g_ = 40): If the government is lax in regulation and fails to effectively supervise the public transit system to provide high quality service, it may be held accountable or penalized by higher authorities or the public. 40 This value should come from the penalties and liability costs that the regulator has suffered from similar cases of policy violations or incidents of public opinion, but due to the difficulty of accessing the inside of the government, this value is based on the comprehensive assessment of the other parameters in this paper. value is based on a comprehensive assessment and analysis of the other parameters in this paper. These parameters are used together in the simulation model, and by adjusting their respective values, we can simulate the impacts of different policies and service qualities on the overall revenues, social benefits, and market size of the transit system.

**Array 1**: R_p_ = 200,C_ph_−C_pl_ = 85,,*B*_*t*_ = 40,*F*_*p*_ = 40,*M*_*p*_ = 30,*C*_*t*_ = 10,*M*_*t*_ = 25,*G*_*c*_ = 20,*T*_*g*_ = 40; based on the analysis of Array 1, the effects of *R*_*p*_,*B*_*t*_,*M*_*t*_,*M*_*p*_,*T*_*g*_ on the process and result of evolutionary game are analyzed.

**Fig 5:**
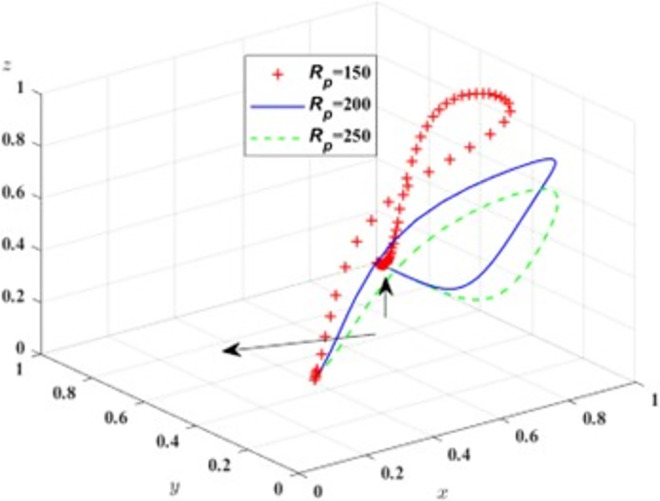
Impact of bus revenue.

**Fig 6:**
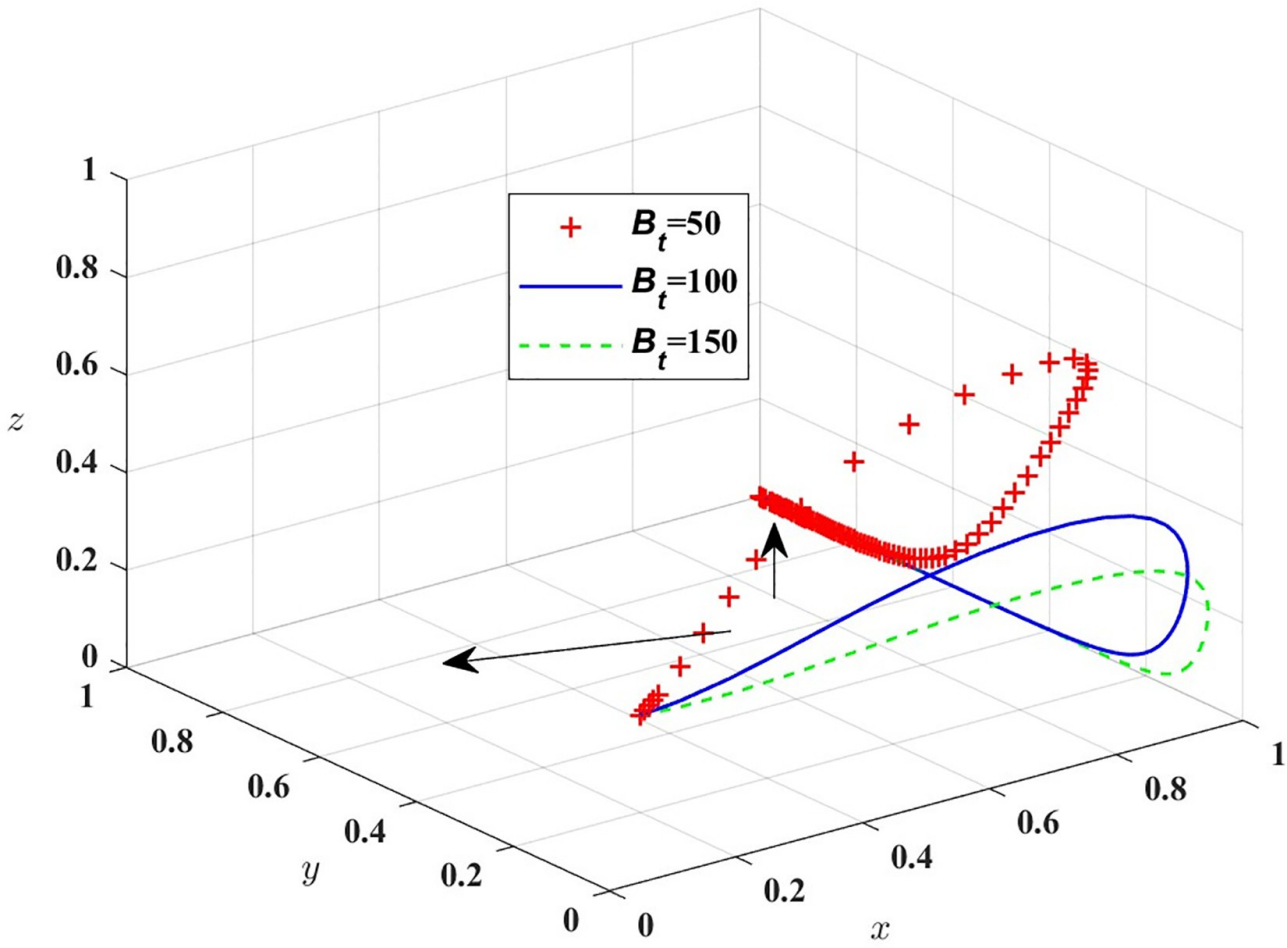
Impact of passenger complaints.

First, in order to analyze the impact of *R*_*p*_ changes on the process and results of the evolutionary game, *R*_*p*_ is assigned the value of *R*_*p*_ = 150, 200, 250, respectively, and the simulation results of replicating the dynamic system of equations evolving over time for 100 times are shown in Fig 5; in order to analyze the impact of *B*_*t*_ changes on the process and results of the evolutionary game, *B*_*t*_ is assigned the value of *B*_*t*_ = 50, 100, 150, respectively, and the simulation results are shown in Fig 6.

From Fig 5, it can be seen that in the process of system evolution until the system stability point is reached, the increase of public transport revenue can accelerate the evolution of public transport to provide high service quality, and with the increase of, the probability of public transport to prov R_p_ ide high service quality rises, and the probability of strict regulation by the government regulator decreases.

**Fig 7:**
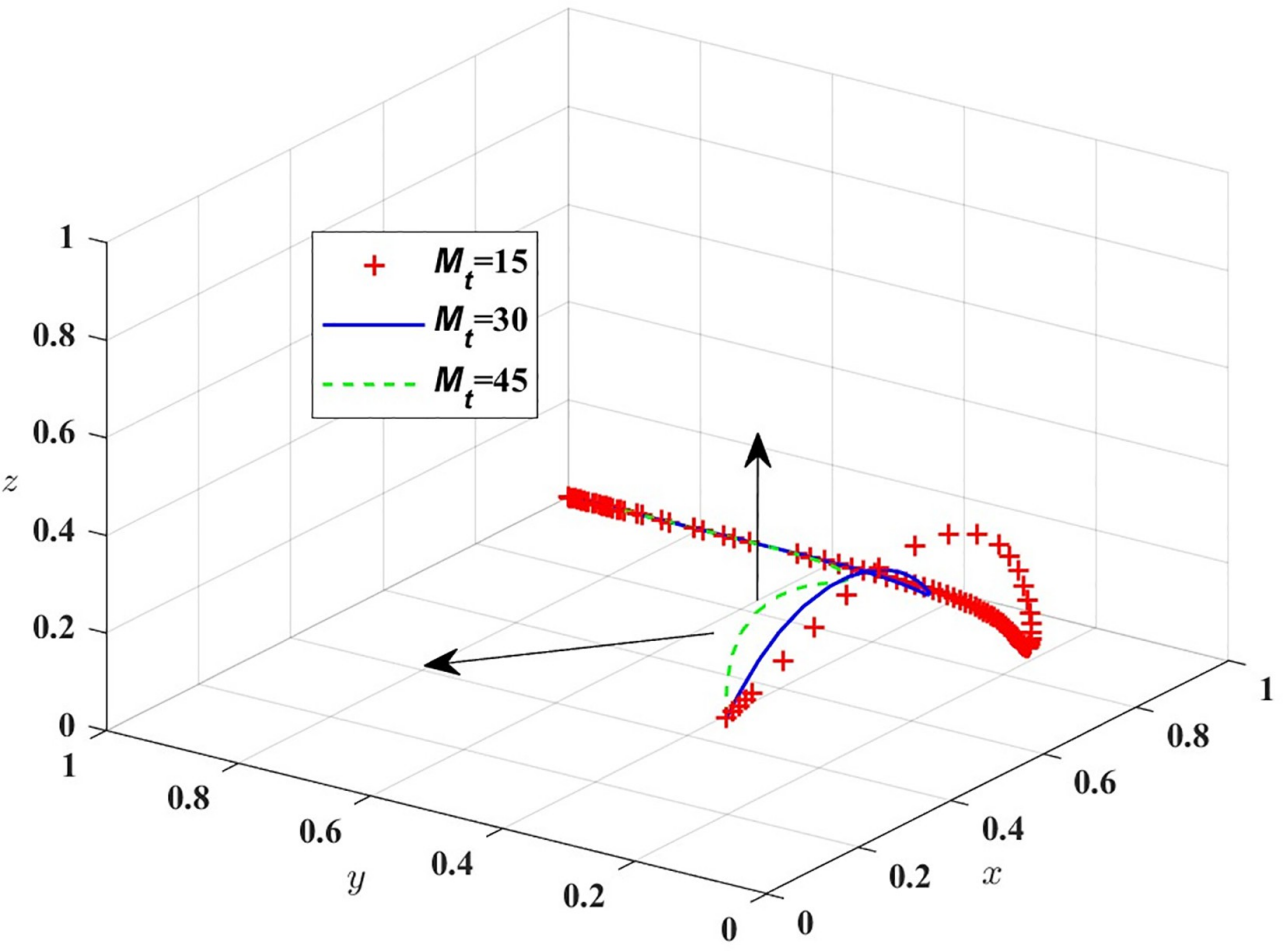
Shows the impact of government rewards for passengers.

Fig 6 shows that in the evolutionary process, with the increase of B_t_, the probability of public transport providing high service quality rises and the probability of passengers not complaining about public transport decreases. The government and other relevant departments can expand its influence through media and other means, or increase the rewards for passengers with valid complaints to cultivate passengers to consciously take social responsibility. Next, *B*_*t*_ = 15, 30, 45 are assigned respectively, and the simulation results are shown in Fig 7.

**Fig 8:**
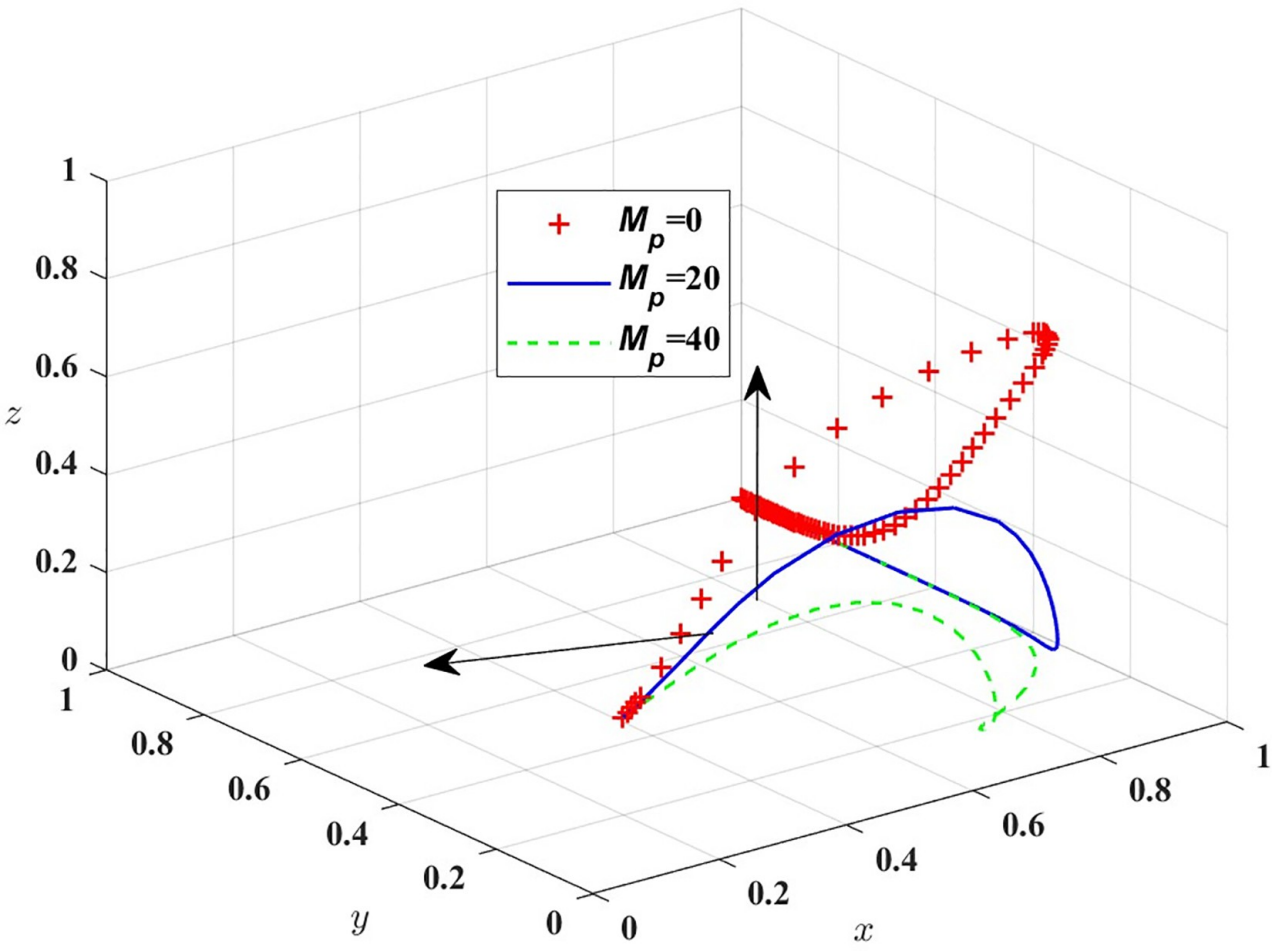
The impact of government rewards on public transportation.

**Fig 9:**
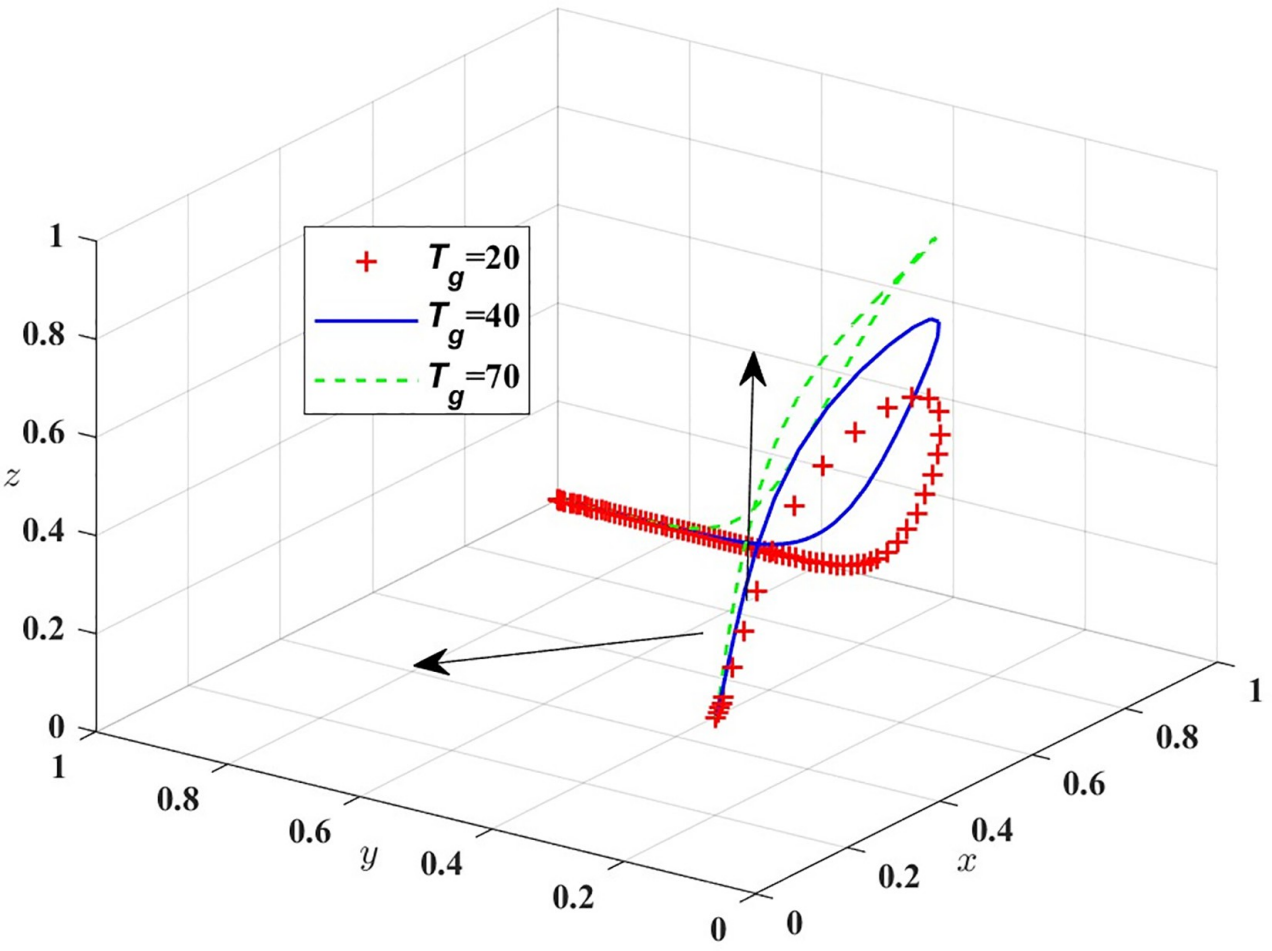
The influence of higher-level penalties on the government.

Fig 7 shows that in the evolution process, the increase of M_t_ will make the probability of strict government regulation decrease, so the government should formulate a reasonable reward and punishment mechanism, and passengers should be appropriately rewarded for the important opinions put forward in the process of travelling by car. Then assigned M_p_ = 0, 20, 40, respectively, the simulation results of replicating the system of dynamic equations evolving over time for 100 times are shown in Fig 8; T_g_ = 20, 40, 70, the simulation results are shown in Fig 9.

Fig 8 shows that in the process of evolutionary stability, the probability of strict supervision by the government will gradually decrease with the increase of M_p_, and the probability of passengers refusing supervision will also increase.

Fig 9 shows that after the evolution probability of public transport providing high-quality service is stable at 1, the increase of T_g_ will increase the probability of strict regulation by the government. It can be seen that although the incentive mechanism of government departments for public transport can promote public transport to provide good services, it is not conducive to the supervision departments to perform their responsibilities.

**Array 2**: R_p_ = 200,C_ph_−C_pl_ = 120, C_p_ = 10,B_t_ = 40,F_p_ = 40,M_p_ = 30,C_t_ = 10, M_t_ = 25,G_c_ = 20,T_g_ = 40; Transit revenue (R_p_ = 200) is the total revenue earned by a transit system under normal operating conditions, usually determined by fare revenue and ridership. This value represents the average revenue that a transit system can earn under normal operating conditions. 200 This value is a reasonable level based on a combination of factors such as ridership and transit fares. This parameter is usually referenced to the average daily actual operating data of local transit systems in China, such as fares, average daily ridership, and operating costs. Cost of High Quality Service (C_ph_)—Cost of Low Quality Service (C_pl_) = 120 indicates the additional cost of providing high quality service over low quality service, which may include vehicle maintenance, facility upgrades, staff training, etc. The difference of 120 is therefore a reasonable level of the cost of providing high quality service over low quality service. Therefore, this 120 difference is an estimate based on historical operating data, budget reports, and equipment upgrade costs, a value typically derived from comparing actual cost differences under different quality levels of service. Stochastic cost (C_p_ = 10) refers to unforeseen expenses or fluctuating costs during operation, such as expenditures for ad hoc repairs, fuel price fluctuations, or other unexpected events. This parameter simulates the impact of uncertainties in actual operations. This value is estimated based on the range of past cost fluctuations.

Reduced revenue from public transit (B_t_ = 40): Loss of revenue due to reduced ridership as a result of poor service quality or other factors, which is usually directly related to passenger satisfaction and willingness to choose public transit. This value is estimated based on actual transit operations. For example, a reasonable value is estimated by counting changes in ridership and revenue loss when service quality changes. Government fine on public transportation (F_p_ = 40): the amount of penalty imposed by the government if the public transportation system fails to meet the service quality standards set by the government, this parameter aims to motivate the public transportation system to improve its service quality through economic means. This value is set according to the actual situation, and in this paper, it is set to 40 based on a combination of revenue and other factors. government incentives for public transportation (M_p_ = 30): the amount of incentives or rewards provided by the government when the public transportation system meets or exceeds the expected quality of service. Government incentives are usually set to motivate public transportation companies to improve service quality, and the value of 30 is an assessment of the actual incentive effect.

Social cost of public transportation to improve low-quality service (C_t_ = 10): the negative impacts and costs to society as a whole when the public transportation system provides low-quality service, such as increased passenger travel time, reduced travel efficiency, and increased pollution. This value is based on a comprehensive assessment and analysis of the parameters set in this paper. Government incentives to passengers (M_t_ = 25): government subsidies or incentives to incentivize more passengers to choose public transport, such as fare concessions, travel subsidies, etc., which are designed to enhance the overall attractiveness of the public transport market. 25 This value is based on the analysis of a comprehensive assessment based on factors such as the amount of subsidies in the policy, as well as the parameters set in this paper. The cost of strict regulation by government departments (G_c_ = 20) refers to the fact that strict regulation requires human, technical, and institutional support, and the parameter value should come from the assessment of the relationship between regulatory intensity and cost inputs, but because this paper is more difficult to access within the government, the value of 20 is set in this paper based on the comprehensive assessment and analysis of the other parameters set in this paper. Penalties for lax regulation (T_g_ = 40): If the government is lax in regulation and fails to effectively supervise the public transit system to provide high quality service, it may be held accountable or penalized by higher authorities or the public. 40 This value should come from the penalties and liability costs that the regulator has suffered from similar cases of policy violations or incidents of public opinion, but due to the difficulty of accessing the inside of the government, this value is based on the comprehensive assessment of the other parameters in this paper. value is based on a comprehensive assessment and analysis of the other parameters in this paper. These parameters are used together in the simulation model, and by adjusting their respective values, we can simulate the impacts of different policies and service qualities on the overall revenues, social benefits, and market size of the transit system.

**Fig 10:**
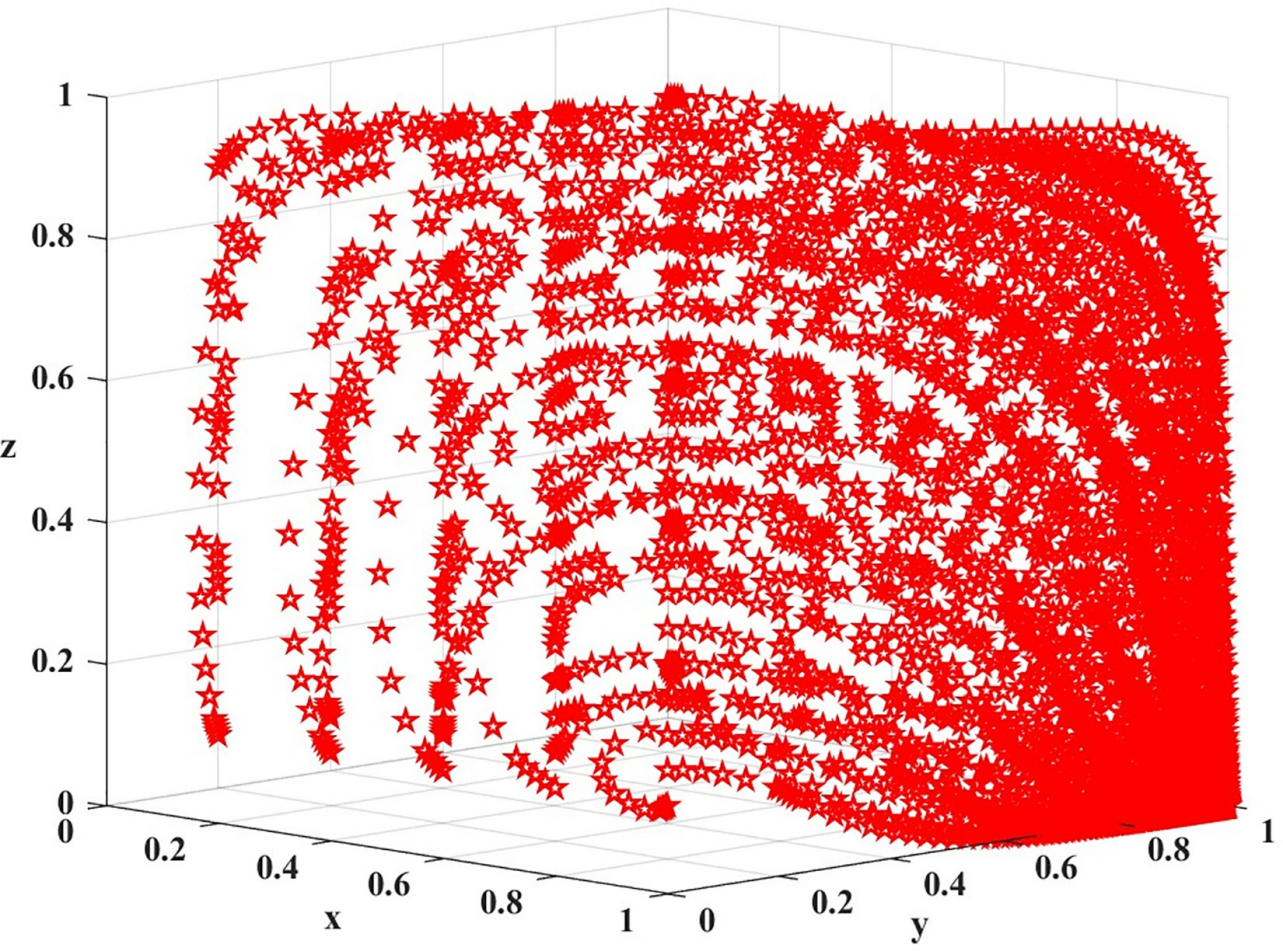
Results of the evolution of array 1 for 100 iterations.

**Fig 11:**
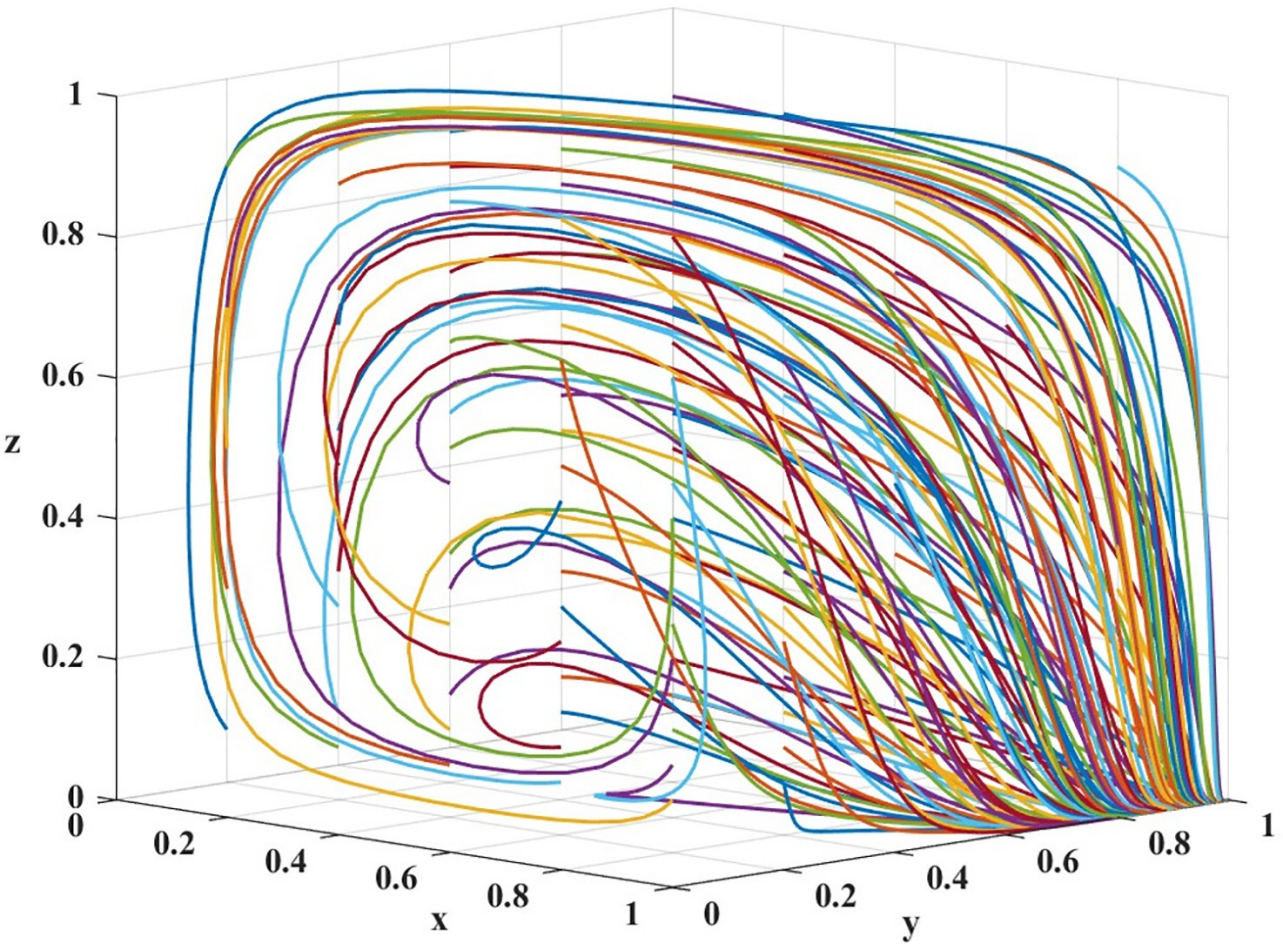
Results of the evolution of array 2 for 100 iterations.

The difference between the cost of high quality services (C_ph_) and the cost of low quality services (C_pl_) represents the additional cost of providing high quality services compared to low quality services. Thus, different values represent different cost differences, and the difference between array 1 and array 2 is the difference between the cost of high quality services and the cost of low quality services. A difference of 85 for array 1 indicates that small improvements are needed to improve service quality, while a difference of 120 for array 2 indicates that greater investment is needed to provide high-quality services. The two sets of values are evolved 100 times over time from different initial strategy combinations, and the results are shown in Figs 10 and 11.

As can be seen in Fig 10, from the simulation results: E_3_ (0,1,0) is the unstable equilibrium point, at this point, there exists only one combination of evolutionary stable strategies (providing high quality service, no complaints from passengers, and loose regulation by government departments).

Fig 11 shows that when $G_{c}{-T}_{g}<0,-C_{t}{-M}_{t},B_{t}{-C}_{ph}{+C}_{pl}{+M}_{p}<0,G_{c}{-F}_{p}{+M}_{t}<0$ is satisfied, there are two evolutionary stability points E_4_(0,0,1) and E_7_(0,1,1). Namely, two evolutionary stability strategy combinations of public transport, passengers and government regulators (providing low-quality service, no complaints, strict supervision) and (providing low-quality service, complaints, strict supervision). Therefore, government regulatory authorities should take into account the interests of all parties and strengthen information construction. Create a good and harmonious environment for the development of public transportation. It can be seen that the simulation analysis is consistent and effective with the conclusion of the strategy stability analysis of all parties, and has certain practical significance for the supervision of public transport to provide high-quality services.

## Conclusions

By constructing a tripartite evolutionary game model between public transport, passengers and government regulators, this paper analyzes the stability of the strategy selection of each party, the stability of the equilibrium strategy combination of the game system and the influence relationship of various elements, and verifies the validity of the conclusions through simulation analyzes. The conclusions are as follows:

First, increasing the government’s incentives or punishment for public transport is conducive to promoting public transport to provide high-quality services to passengers, but increasing the government’s incentives for public transport to provide high-quality services is not conducive to the government’s regulators to better perform their regulatory responsibilities; In the process of evolution, the probability of public transport providing high quality of service rises as the rate of complaints from passengers and the strict supervision rate by government regulators increases. The government plays an important role in incentivising the public transport to provide high-quality services. Although the incentive and punishment mechanism is crucial to guide the positive behavior of the public transport, over-incentive may lead to the reduction of the government’s constraint on its own behavior. Therefore, it is particularly crucial to develop a reasonable incentive and punishment mechanism. Moreover, the accountability of the higher level government to the public transport supervision department is of great significance to the provision of high-quality public transport services.

Second, the probability of travelling passengers making complaints is negatively correlated with the revenue of public transport, and positively correlated with the amount of punishment of government regulators on public transport, and the amount of incentives for passengers. In the evolution process, the behavior of passengers not to complain will increase with the strict supervision rate of government regulatory authorities or the probability of public transport providing high-quality services. For public transport passengers, more active and proactive in fulfilling the basic obligations of citizens is the key to achieve the harmonious and stable development of public transport. In the process of taking public transport, passengers should supervise the services provided by the public transport, in order to make the public transport better constraints on their own behavior, the passengers should be constrained on their own behavior.

Third, the probability of strict regulation by government regulators is positively correlated with government fines for public transport, and negatively correlated with government regulators’ rewards for travelling passengers. In the process of evolution, strict supervision by government regulators will decrease with the increase of the probability that the public transport provides high-quality service or the number of passengers does not complain. The Public transport should comply with the rules of the public transport system and use the Internet to connect the data between the platform and passengers. The drivers, conductors and ticket checkers should be trained to improve their professionalism, and the service quality can be improved by expanding the media disclosure.

## Supporting information

S1 File Matlab program for obtaining the eigenvalues of the 13 equilibrium. (DOCX)

S2 File Matlab program for plotting Fig 10. (DOCX)
