# Supplementary material for: Evolutionary game and simulation study of public transport under government incentive and punishment mechanism
Source: PLoS One. 2024 Oct 1;19(10):e0311286. doi: 10.1371/journal.pone.0311286 (PMC11444410; doi:10.1371/journal.pone.0311286)
Supplement: S1 File — (DOCX) [file pone.0311286.s001.docx]

%%MATLAB求解三阶雅克比矩阵的特征根程序

%注释符过多可以在学完之后删除，一定要在英文输入法下输入标点、字母

%%数值变量

clc;clear;

A = [1 0 0;0 2 0;0 0 3]; %将这里的数字改为你想要的数字，其他勿动

[V,R] = eig(A),DA=det(A),IA=inv(A), %V是列向量组成的矩阵，R对角元素是特征根，DA是行列式，IA是逆矩阵，这行的逗号不能动

clc;clear;

A = [1 2 4;

4 0 7;

9 1 3];

[V,R] = eig(A),DA=det(A),IA=inv(A),

%%字符变量

clc;clear;

syms a11 a12 a13; %中间一定是空格隔开，字符变量一定要先定义再使用，前后保持一致

A = [a11 0 0;

0 a12 0;

0 0 a13]; %将这里的数字改为你想要的数字，其他勿动

[V,R] = eig(A),DA=det(A),IA=inv(A), %V是列向量组成的矩阵，R对角元素是特征根，DA是行列式，IA是逆矩阵，这行的逗号不能动

%%

%政府奖惩机制下药品安全质量监管三方演化博弈及仿真分析-雅克比矩阵特征值求解

%均衡点E1（找R）

clc;clear;

syms x y z Cph Cpl Cp Bt Rp Fp Mp Mt Ft Ct Tg Cg;

x=0,y=0,z=0; %均衡点E1

A = [(2*x-1)*(Cph-Cpl-Cp-Bt-y*(Rp-Bt)-z*(Fp+Mp)) x*(x-1)*(Bt-Rp) x*(x-1)*(-Fp-Mp);

y*(y-1)*(-Bt+Mt) (2*y-1)*((1-x)*(Bt-Mt)-z*(Ft+Mt)-Ct) y*(y-1)*(-Ft-Mt);

z*(z-1)*(Mp+Fp+Tg-y*Tg) z*(z-1)*(Mt+Ft+Tg-x*Tg) (2*z-1)*(Cg-Fp-Ft-Tg+x*(Mp+Fp+Tg)+y*(Mt+Ft+Tg)-x*y*Tg)];

[V,R] = eig(A),DA=det(A),IA=inv(A),

%均衡点E2（找R）

clc;clear;

syms x y z Cph Cpl Cp Bt Rp Fp Mp Mt Ft Ct Tg Cg;

x=1,y=0,z=0; %均衡点E2

A = [(2*x-1)*(Cph-Cpl-Cp-Bt-y*(Rp-Bt)-z*(Fp+Mp)) x*(x-1)*(Bt-Rp) x*(x-1)*(-Fp-Mp);

y*(y-1)*(-Bt+Mt) (2*y-1)*((1-x)*(Bt-Mt)-z*(Ft+Mt)-Ct) y*(y-1)*(-Ft-Mt);

z*(z-1)*(Mp+Fp+Tg-y*Tg) z*(z-1)*(Mt+Ft+Tg-x*Tg) (2*z-1)*(Cg-Fp-Ft-Tg+x*(Mp+Fp+Tg)+y*(Mt+Ft+Tg)-x*y*Tg)];

[V,R] = eig(A),DA=det(A),IA=inv(A),

%均衡点E3（找R）

clc;clear;

syms x y z Cph Cpl Cp Bt Rp Fp Mp Mt Ft Ct Tg Cg;

x=0,y=1,z=0; %均衡点E3

A = [(2*x-1)*(Cph-Cpl-Cp-Bt-y*(Rp-Bt)-z*(Fp+Mp)) x*(x-1)*(Bt-Rp) x*(x-1)*(-Fp-Mp);

y*(y-1)*(-Bt+Mt) (2*y-1)*((1-x)*(Bt-Mt)-z*(Ft+Mt)-Ct) y*(y-1)*(-Ft-Mt);

z*(z-1)*(Mp+Fp+Tg-y*Tg) z*(z-1)*(Mt+Ft+Tg-x*Tg) (2*z-1)*(Cg-Fp-Ft-Tg+x*(Mp+Fp+Tg)+y*(Mt+Ft+Tg)-x*y*Tg)];

[V,R] = eig(A),DA=det(A),IA=inv(A),

%均衡点E4（找R）

clc;clear;

syms x y z Cph Cpl Cp Bt Rp Fp Mp Mt Ft Ct Tg Cg;

x=0,y=0,z=1; %均衡点E4

A = [(2*x-1)*(Cph-Cpl-Cp-Bt-y*(Rp-Bt)-z*(Fp+Mp)) x*(x-1)*(Bt-Rp) x*(x-1)*(-Fp-Mp);

y*(y-1)*(-Bt+Mt) (2*y-1)*((1-x)*(Bt-Mt)-z*(Ft+Mt)-Ct) y*(y-1)*(-Ft-Mt);

z*(z-1)*(Mp+Fp+Tg-y*Tg) z*(z-1)*(Mt+Ft+Tg-x*Tg) (2*z-1)*(Cg-Fp-Ft-Tg+x*(Mp+Fp+Tg)+y*(Mt+Ft+Tg)-x*y*Tg)];

[V,R] = eig(A),DA=det(A),IA=inv(A),

%均衡点E5（找R）

clc;clear;

syms x y z Cph Cpl Cp Bt Rp Fp Mp Mt Ft Ct Tg Cg;

x=1,y=1,z=0; %均衡点E5

A = [(2*x-1)*(Cph-Cpl-Cp-Bt-y*(Rp-Bt)-z*(Fp+Mp)) x*(x-1)*(Bt-Rp) x*(x-1)*(-Fp-Mp);

y*(y-1)*(-Bt+Mt) (2*y-1)*((1-x)*(Bt-Mt)-z*(Ft+Mt)-Ct) y*(y-1)*(-Ft-Mt);

z*(z-1)*(Mp+Fp+Tg-y*Tg) z*(z-1)*(Mt+Ft+Tg-x*Tg) (2*z-1)*(Cg-Fp-Ft-Tg+x*(Mp+Fp+Tg)+y*(Mt+Ft+Tg)-x*y*Tg)];

[V,R] = eig(A),DA=det(A),IA=inv(A),

%均衡点E6（找R）

clc;clear;

syms x y z Cph Cpl Cp Bt Rp Fp Mp Mt Ft Ct Tg Cg;

x=1,y=0,z=1; %均衡点E6

A = [(2*x-1)*(Cph-Cpl-Cp-Bt-y*(Rp-Bt)-z*(Fp+Mp)) x*(x-1)*(Bt-Rp) x*(x-1)*(-Fp-Mp);

y*(y-1)*(-Bt+Mt) (2*y-1)*((1-x)*(Bt-Mt)-z*(Ft+Mt)-Ct) y*(y-1)*(-Ft-Mt);

z*(z-1)*(Mp+Fp+Tg-y*Tg) z*(z-1)*(Mt+Ft+Tg-x*Tg) (2*z-1)*(Cg-Fp-Ft-Tg+x*(Mp+Fp+Tg)+y*(Mt+Ft+Tg)-x*y*Tg)];

[V,R] = eig(A),DA=det(A),IA=inv(A),

%均衡点E7（找R）

clc;clear;

syms x y z Cph Cpl Cp Bt Rp Fp Mp Mt Ft Ct Tg Cg;

x=0,y=1,z=1; %均衡点E7

A = [(2*x-1)*(Cph-Cpl-Cp-Bt-y*(Rp-Bt)-z*(Fp+Mp)) x*(x-1)*(Bt-Rp) x*(x-1)*(-Fp-Mp);

y*(y-1)*(-Bt+Mt) (2*y-1)*((1-x)*(Bt-Mt)-z*(Ft+Mt)-Ct) y*(y-1)*(-Ft-Mt);

z*(z-1)*(Mp+Fp+Tg-y*Tg) z*(z-1)*(Mt+Ft+Tg-x*Tg) (2*z-1)*(Cg-Fp-Ft-Tg+x*(Mp+Fp+Tg)+y*(Mt+Ft+Tg)-x*y*Tg)];

[V,R] = eig(A),DA=det(A),IA=inv(A),

%均衡点E8（找R）

clc;clear;

syms x y z Cph Cpl Cp Bt Rp Fp Mp Mt Ft Ct Tg Cg;

x=1,y=1,z=1; %均衡点E8

A = [(2*x-1)*(Cph-Cpl-Cp-Bt-y*(Rp-Bt)-z*(Fp+Mp)) x*(x-1)*(Bt-Rp) x*(x-1)*(-Fp-Mp);

y*(y-1)*(-Bt+Mt) (2*y-1)*((1-x)*(Bt-Mt)-z*(Ft+Mt)-Ct) y*(y-1)*(-Ft-Mt);

z*(z-1)*(Mp+Fp+Tg-y*Tg) z*(z-1)*(Mt+Ft+Tg-x*Tg) (2*z-1)*(Cg-Fp-Ft-Tg+x*(Mp+Fp+Tg)+y*(Mt+Ft+Tg)-x*y*Tg)];

[V,R] = eig(A),DA=det(A),IA=inv(A),

%均衡点E9（找R）

clc;clear;

syms x y z Cph Cpl Cp Bt Rp Fp Mp Mt Ft Ct Tg Cg y1 z1;

x=0,y=y1,z=z1; %均衡点E9

A = [(2*x-1)*(Cph-Cpl-Cp-Bt-y*(Rp-Bt)-z*(Fp+Mp)) x*(x-1)*(Bt-Rp) x*(x-1)*(-Fp-Mp);

y*(y-1)*(-Bt+Mt) (2*y-1)*((1-x)*(Bt-Mt)-z*(Ft+Mt)-Ct) y*(y-1)*(-Ft-Mt);

z*(z-1)*(Mp+Fp+Tg-y*Tg) z*(z-1)*(Mt+Ft+Tg-x*Tg) (2*z-1)*(Cg-Fp-Ft-Tg+x*(Mp+Fp+Tg)+y*(Mt+Ft+Tg)-x*y*Tg)];

[V,R] = eig(A),DA=det(A),IA=inv(A),

%均衡点E10（找R）

clc;clear;

syms x y z Cph Cpl Cp Bt Rp Fp Mp Mt Ft Ct Tg Cg x1 z2;

x=x1,y=0,z=z2; %均衡点E10

A = [(2*x-1)*(Cph-Cpl-Cp-Bt-y*(Rp-Bt)-z*(Fp+Mp)) x*(x-1)*(Bt-Rp) x*(x-1)*(-Fp-Mp);

y*(y-1)*(-Bt+Mt) (2*y-1)*((1-x)*(Bt-Mt)-z*(Ft+Mt)-Ct) y*(y-1)*(-Ft-Mt);

z*(z-1)*(Mp+Fp+Tg-y*Tg) z*(z-1)*(Mt+Ft+Tg-x*Tg) (2*z-1)*(Cg-Fp-Ft-Tg+x*(Mp+Fp+Tg)+y*(Mt+Ft+Tg)-x*y*Tg)];

[V,R] = eig(A),DA=det(A),IA=inv(A),

%均衡点E12（找R）

clc;clear;

syms x y z Cph Cpl Cp Bt Rp Fp Mp Mt Ft Ct Tg Cg x2 y2;

x=x2,y=y2,z=0; %均衡点E12

A = [(2*x-1)*(Cph-Cpl-Cp-Bt-y*(Rp-Bt)-z*(Fp+Mp)) x*(x-1)*(Bt-Rp) x*(x-1)*(-Fp-Mp);

y*(y-1)*(-Bt+Mt) (2*y-1)*((1-x)*(Bt-Mt)-z*(Ft+Mt)-Ct) y*(y-1)*(-Ft-Mt);

z*(z-1)*(Mp+Fp+Tg-y*Tg) z*(z-1)*(Mt+Ft+Tg-x*Tg) (2*z-1)*(Cg-Fp-Ft-Tg+x*(Mp+Fp+Tg)+y*(Mt+Ft+Tg)-x*y*Tg)];

[V,R] = eig(A),DA=det(A),IA=inv(A),

%均衡点E13（找R）

clc;clear;

syms x y z Cph Cpl Cp Bt Rp Fp Mp Mt Ft Ct Tg Cg x3 y3;

x=x3,y=y3,z=1; %均衡点E13

A = [(2*x-1)*(Cph-Cpl-Cp-Bt-y*(Rp-Bt)-z*(Fp+Mp)) x*(x-1)*(Bt-Rp) x*(x-1)*(-Fp-Mp);

y*(y-1)*(-Bt+Mt) (2*y-1)*((1-x)*(Bt-Mt)-z*(Ft+Mt)-Ct) y*(y-1)*(-Ft-Mt);

z*(z-1)*(Mp+Fp+Tg-y*Tg) z*(z-1)*(Mt+Ft+Tg-x*Tg) (2*z-1)*(Cg-Fp-Ft-Tg+x*(Mp+Fp+Tg)+y*(Mt+Ft+Tg)-x*y*Tg)];

[V,R] = eig(A),DA=det(A),IA=inv(A),
