# Supplementary material for: Evolutionary game and simulation study of public transport under government incentive and punishment mechanism
Source: PLoS One. 2024 Oct 1;19(10):e0311286. doi: 10.1371/journal.pone.0311286 (PMC11444410; doi:10.1371/journal.pone.0311286)
Supplement: S2 File — (DOCX) [file pone.0311286.s002.docx]

%% 三维x-y-z数组

%不同初始策略组合演化图

%%

%图10，数组1

clc,clear;

figure(10);

Rp=200,Cph=120,Cpl=0,Cp=10,Bt=40,Fp=40,Mp=30,Ct=10,Ft=50,Mt=25,Gc=20,Tg=40;

for i=0.1:0.2:1

for j=0.1:0.2:1

for k=0.1:0.2:1

[t,y]=ode45(@(t,y) yaopin(t,y,Rp,Cph,Cpl,Cp,Bt,Fp,Mp,Ct,Ft,Mt,Gc,Tg),[0 100],[i j k]);

%plot3(y(:,1),y(:,2),y(:,3),'linewidth',1);

plot3(y(:,1),y(:,2),y(:,3),'rp','linewidth',1); %把颜色改为红色，线型改为五角星。

set(gca,'XTick',[0:0.2:1],'YTick',[0:0.2:1],'ZTick',[0:0.2:1])

hold on

axis([0 1 0 1 0 1])

view([45 10])

end

end

end

grid on

hold on

xlabel('x','Rotation',0);

ylabel('y','Rotation',0);

zlabel('z','Rotation',360,'position',[0 0 1.05]);

title('图 10 数组1演化100次结果','FontWeight','bold','position',[1 0 -0.13])
